# Progranulin-dependent repair function of regulatory T cells drives bone-fracture healing

**DOI:** 10.1172/JCI180679

**Published:** 2024-11-07

**Authors:** Ruiying Chen, Xiaomeng Zhang, Bin Li, Maurizio S. Tonetti, Yijie Yang, Yuan Li, Beilei Liu, Shujiao Qian, Yingxin Gu, Qingwen Wang, Kairui Mao, Hao Cheng, Hongchang Lai, Junyu Shi

**Affiliations:** 1 Department of Oral and Maxillofacial Implantology, Shanghai PerioImplant Innovation Center, Shanghai Ninth People’s Hospital, Shanghai Jiao Tong University School of Medicine, Shanghai Jiao Tong University, National Center for Stomatology, National Clinical Research Center for Oral Diseases, Shanghai Key Laboratory of Stomatology, Shanghai, China.; 2 Center for Immune-Related Diseases at Shanghai Institute of Immunology, Department of Respiratory and Critical Care Medicine of Ruijin Hospital, Department of Thoracic Surgery of Ruijin Hospital, Department of Immunology and Microbiology, Shanghai Jiao Tong University School of Medicine, Shanghai, China.; 3 Department of Integrated TCM & Western Medicine, Shanghai Skin Disease Hospital, School of Medicine, Tongji University, Shanghai, China.; 4 Department of Thoracic Surgery, Shanghai Pulmonary Hospital, Tongji University, Shanghai, China.; 5 Department of Oncology, Department of Hepatobiliary Surgery, The First Affiliated Hospital of Anhui Medical University, Hefei, China.; 6 Shenzhen Key Laboratory of Immunity and Inflammatory Diseases, Shenzhen, Guangdong, China.; 7 Department of Rheumatism and Immunology, Peking University Shenzhen Hospital, Guangdong, China.; 8 State Key Laboratory of Cellular Stress Biology, Innovation Center for Cell Signaling Network, School of Life Sciences, Xiamen University, Xiamen, China.; 9 Center for Immune-Related Diseases at Shanghai Institute of Immunology, Department of Respiratory and Critical Care Medicine of Ruijin Hospital, Department of Thoracic Surgery of Ruijin Hospital, Department of Immunology and Microbiology, Shanghai Jiao Tong University School of Medicine, Shanghai, China.; 10 Department of Rheumatism and Immunology, Peking University Shenzhen Hospital, Guangdong, China.; 11 Center for Cancer Immunology Research, Shenzhen Institutes of Advanced Technology, Chinese Academy of Sciences, Shenzhen, Guangdong, China.

**Keywords:** Bone biology, Bone disease, Bone marrow

## Abstract

Local immunoinflammatory events instruct skeletal stem cells (SSCs) to repair/regenerate bone after injury, but mechanisms are incompletely understood. We hypothesized that specialized Tregs are necessary for bone repair and interact directly with SSCs through organ-specific messages. Both in human patients with bone fracture and a mouse model of bone injury, we identified a bone injury–responding Treg subpopulation with bone-repair capacity marked by CCR8. Local production of CCL1 induced a massive migration of CCR8^+^ Tregs from periphery to the injury site. Depending on secretion of progranulin (PGRN), a protein encoded by the granulin (*Grn*) gene, CCR8^+^ Tregs supported the accumulation and osteogenic differentiation of SSCs and thereby bone repair. Mechanistically, we revealed that CCL1 enhanced expression levels of basic leucine zipper ATF-like transcription factor (BATF) in CCR8^+^ Tregs, which bound to the *Grn* promoter and increased *Grn* translational output and then PGRN secretion. Together, our work provides a new perspective in osteoimmunology and highlights possible ways of manipulating Treg signaling to enhance bone repair and regeneration.

## Abstract 2

Regulatory T cells secreted PGRN to drive bone fracture healing via interaction with skeletal stem cells in a CCR8-CCL1 dependent manner.

## Graphical abstract

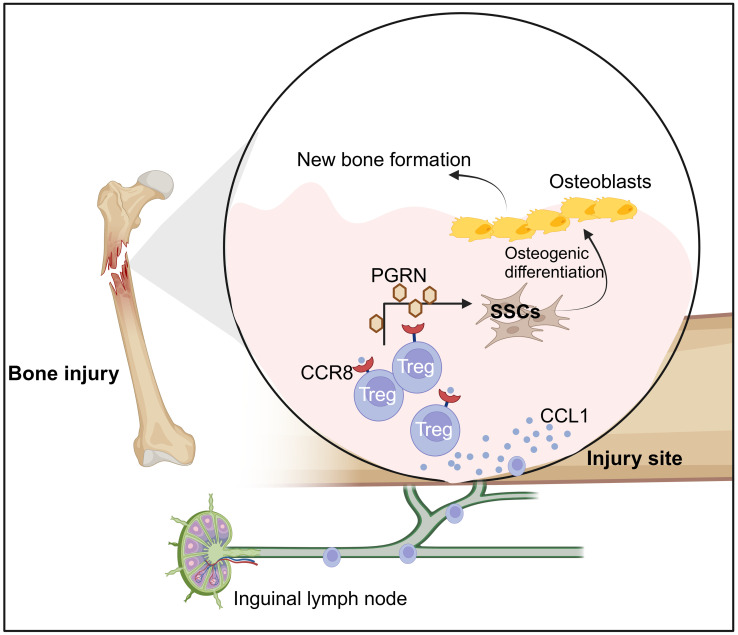

## Introduction

Bone fractures are a serious public health issue due to their high prevalence and serious consequences (178 million cases in 2019) (1). They may heal with complications (nonunion) and frequently require surgical intervention, especially in aging subjects. Bone repair depends on the highly coordinated mobilization, proliferation, and differentiation of skeletal stem cells (SSCs) and their progeny within the injury site (2, 3). After an injury, quiescent stem cells receive signals from the local environment to activate their bone-repair function (3, 4). However, previous bone-repair studies (5–7) were mainly performed with mesenchymal stem cells: a heterogeneous cell population with varying differentiating potential and including cells from other lineages rather than uniform purified SSCs. Thus, knowledge of the exact cues within the local environment instructing SSCs toward bone repair is limited. Recently, through a combination of FACS and lineage reporter mice, highly purified SSCs, the highly selective and homogeneous cell population responsible for bone organ development, growth, and regeneration/repair, have been identified (8, 9). This enables direct investigation of the instructing mechanism of SSC function during bone repair.

After bone injury, immune cells first trigger inflammation and later produce the necessary antiinflammatory signals that allow SSCs to initiate bone repair (10, 11). Besides the established roles of macrophages and neutrophils (5, 12, 13), multiple lines of evidence point to a possible role of Tregs in bone repair and homeostasis (14–16). Decreased Treg accumulation has been observed in patients with nonhealing fractures (17–20), like AIDS patients (19, 20). Additionally, parathyroid hormone–induced (PTH-induced) bone formation was impaired by Treg ablation (21, 22). The mechanism(s) of these observations have yet to be elucidated, and it is unclear if Tregs can directly affect SSC function.

In addition, Tregs are a vital component of the tissue stem-cell niche (23, 24): they support stem-cell function, maintain tissue homeostasis, and promote tissue repair in multiple organs, including lung (25), cardiac muscle (26, 27), skeletal muscle (28), skin (29), and brain (30). The bone marrow (BM) is also a known reservoir for Tregs (14).

We hypothesized that Tregs are necessary for bone repair and interact directly with SSCs. Through using a mouse model of bone injury combined with single-cell RNA sequencing (scRNA-Seq) of intramedullary canal tissue samples obtained from human patients with bone fracture (31), we showed that bone repair required the accumulation of a unique Treg subpopulation with bone-repair signature and marked by CCR8 at the injury site. These cells secreted progranulin (PGRN), encoded by the granulin (*Grn*) gene, to promote SSC accumulation, osteogenic differentiation, and then bone repair. Moreover, local production of CCL1 recruited the CCR8^+^ Tregs from periphery to the injury site and promoted their secretion of PGRN through enhanced transcriptional activity of *Grn* induced by basic leucine zipper ATF-like transcription factor (BATF). Collectively, our study elucidated an immune-related bone-repair mechanism mediated by CCR8^+^ Tregs.

## Results

### Tregs accumulate at the bone injury site and are required for bone repair.

**Figure 1:**
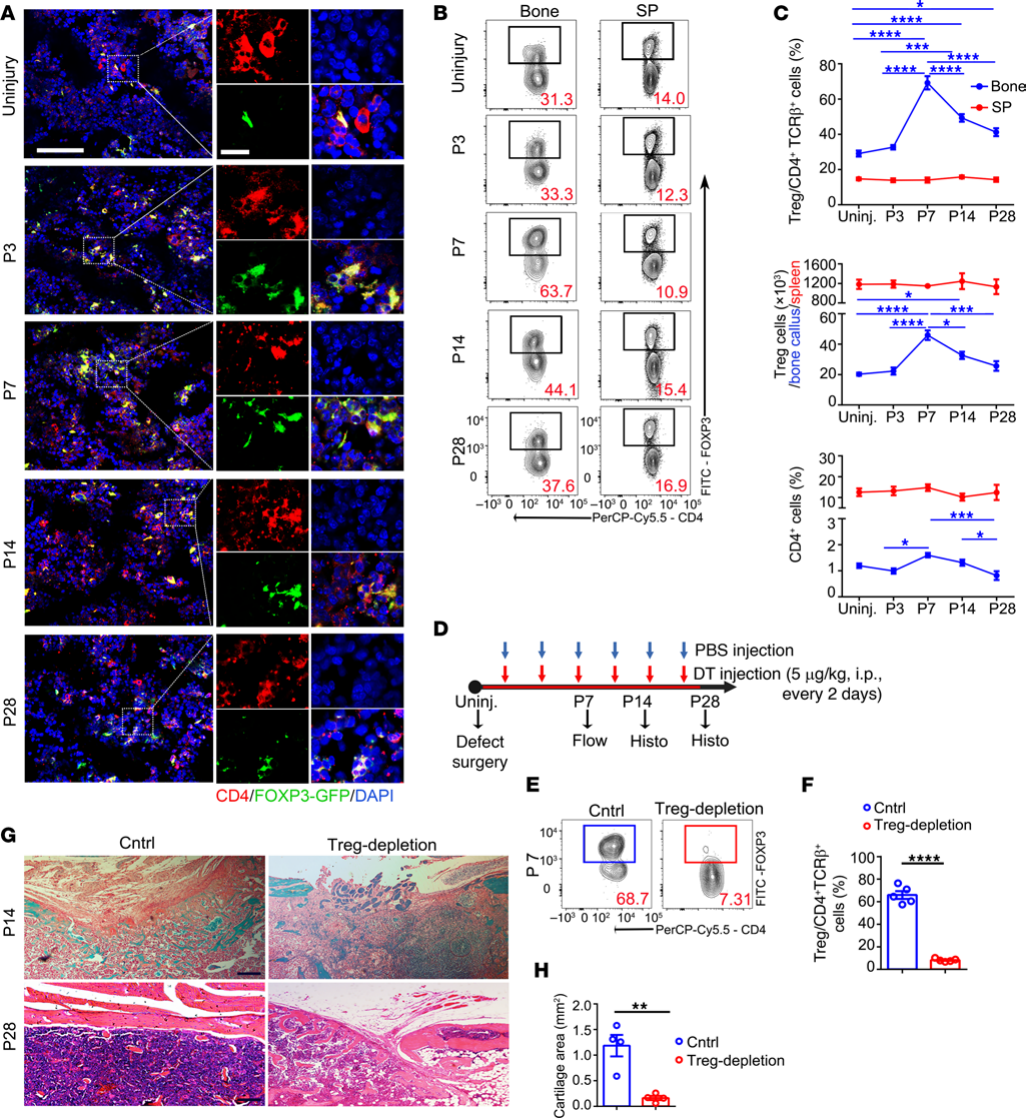
Treg accumulation in injured bone tissue enables bone repair. (**A**) Representative images of colocalization of CD4 (red) and FOXP3-GFP (green) in uninjured/injured bone tissue on day 0 (uninjury), day 3, day 7, day 14, and day 28 after operation. Scale bars: 100 μm (left); 10 μm (right). (**B**) Representative flow cytometry analysis of Tregs from callus tissue on day 0 (uninjury), day 3, day 7, day 14, and day 28 after operation. The numbers indicate the proportion of Tregs in the frame. (**C**) The proportions of Tregs among CD4^+^TCRβ^+^ cells, the numbers of Tregs, and the proportions of CD4^+^ cells at indicated time points in bone callus tissue (blue) and in SP (red). *n* = 4–5 per group. (**D**) Diagram illustration of Treg depletion by DT injection after bone-defect surgery. (**E**) Representative flow cytometry graphs of Treg proportions in control group (Cntrl) and Treg-depletion group. The numbers indicate the proportion of Tregs in the frame. (**F**) The proportions of Tregs among CD4^+^TCRβ^+^ cells. *n* = 5 per group. (**G**) Safranin O staining of injured bone tissues from the control group and Treg-depletion group. Scale bars: 200 μm. (**H**) The Safranin O images were analyzed using Image J, version 1.48. *n* = 4 per group. (**I**) H&E staining of injured bone tissues from the control group and Treg-depletion group. Scale bars: 200 μm. All data are represented as mean ± SEM. *P ≤ 0.05; **P ≤ 0.01; ***P ≤ 0.005; ****P ≤ 0.001, as determined by unpaired 2-tailed Student’s *t* test (**F** and **H**) or 1-way ANOVA with Bonferroni’s multiple-comparisons test (**C**).

We first observed Tregs’ involvement in bone healing after standardized drill-hole surgery in the femur. During the bone-repair process (Supplemental Figure 1A; supplemental material available online with this article; https://doi.org/10.1172/JCI180679DS1), Tregs significantly accumulated at the injury site, as indicated by more abundant positive staining of FOXP3-GFP from 3 days after surgery (Figure 1A and Supplemental Figure 1B). Flow cytometry analysis of the cells harvested from the wound/callus tissue (including nearby BM) (Figure 1, B and C, and Supplemental Figure 1, C and D) showed that the proportions of Tregs in the injury tissue increased from day 3 after injury and peaked on day 7 when they accounted for 69.2% ± 7.5% of the CD4^+^TCRβ^+^ cells. The absolute number of Tregs within the injury site also showed marked increase on day 7, remained high, and slowly returned to normal 1 month after injury. No significant changes in Treg proportions and numbers were observed in the spleen (SP) of the same mice over the time course (Figure 1, B and C).

To verify the critical role of Tregs in bone repair, we selectively depleted Tregs by injecting diphtheria toxin (DT) into *Foxp3*-DTR (DT receptor) transgenic mice (Treg-depletion group) after bone injury (Figure 1D). Untreated *Foxp3*-DTR transgenic mice were used as a control group. Seven days after DT administration, over 85% of CD4^+^FOXP3^+^ cells were effectively depleted from the BM compared with untreated *Foxp3*-DTR mice (Figure 1, E and F). In the Treg-depletion group, safranin O staining revealed the reduction of deposited bone matrix and cartilage tissue in the injury lesion on day 14 (Figure 1, G and H). H&E staining results showed nonunion with primarily fibrous tissue around the injury site in the Treg-depletion group on day 28, whereas the control group exhibited completed union (Figure 1I). We also verified the results in a femur fracture model in mice. In the Treg-depletion group, the absence of callus and nonunion was observed, but in the controls, fracture healing was observed in all cases (Supplemental Figure 2).

As previous studies have indicated a tight relationship between Tregs and osteoclasts (16, 32), we also evaluated if Treg depletion changed the numbers or distribution of osteoclasts in the BM. Tartrate-resistant acid phosphatase (TRAP) staining showed no significant difference in the osteoclast number between the control and Treg-depletion groups (Supplemental Figure 3). These results indicated that increased accumulation of Tregs is required during bone repair.

### Tregs at the bone injury site are essential for promoting SSC accumulation and osteogenic differentiation.

**Figure 2:**
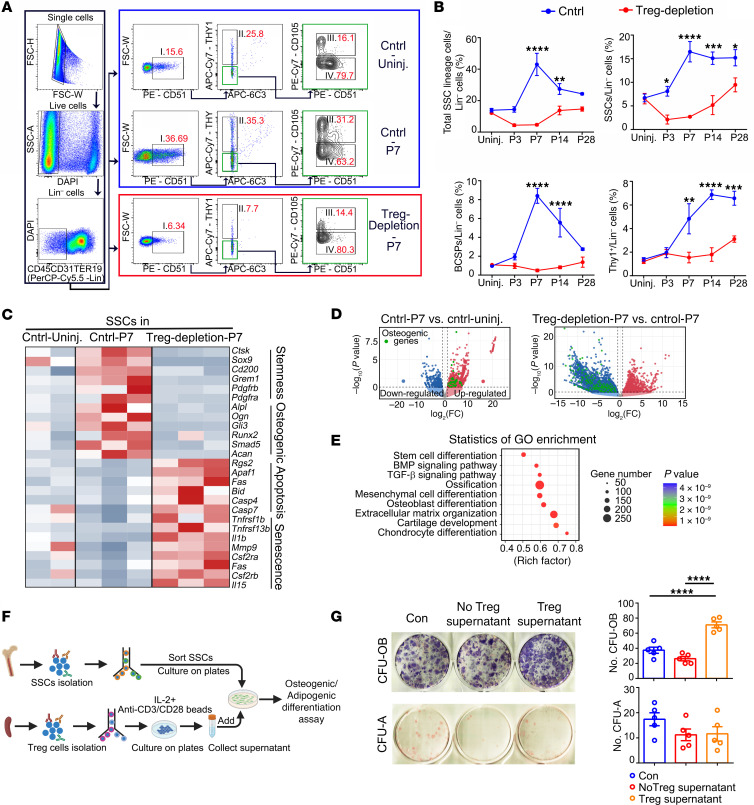
Tregs promote SSC accumulation and osteogenic differentiation. (**A**) Representative flow cytometry analysis images of SSC lineage cells. (**B**) The percentages of total SSC lineage cells, SSCs, BCSPs, and Thy1^+^ cells within total Lin^–^ cells from control and Treg-depletion groups at indicated time points. *n* = 4 per group. (**C**) Heatmap of a selected list of differentially expressed transcripts among SSCs isolated 7 days after injury (Cntrl-P7), SSCs before injury (Cntrl-Uninj.) and SSCs from Treg-depletion group 7 days after injury (Treg-depletion-P7). *n* = 2–3 per group. (**D**) Volcano plot representation of a comparison of gene-expression profiles between SSCs from Cntrl-P7 group and Cntrl-Uninj. group or SSCs from Treg-depletion-P7 group and Cntrl-P7 group. Green dots show osteogenic genes. (**E**) GO analysis revealing GO enrichment of the biological processes enriched in SSCs from Cntrl-P7 group compared with SSCs from Treg-depletion-P7 group. (**F**) Diagram of the experimental protocol showing the isolation of Tregs from the SP and SSCs from the bone tissue of the same mice. The SSCs were cultured with the Treg-conditioned medium and subjected to osteogenic/adipogenic differentiation assays. (**G**) Quantification of colonies of osteogenic (CFU-OB) and adipogenic (CFU-Adipo) assays showing the differentiation results of control group (SSCs cultured without adding Treg culture medium), no Treg supernatant group (SSCs cultured with adding fresh Treg culture medium), and Treg supernatant group (SSCs cultured with adding supernatant collected from cultured Treg dishes). *n* = 5 per group. All data are represented as mean ± SEM. *P ≤ 0.05, **P ≤ 0.01, ***P ≤ 0.005, ****P ≤ 0.001, as determined by 2-way ANOVA with Bonferroni’s multiple-comparisons test (**B**) or 1-way ANOVA with Bonferroni’s multiple-comparisons test (**G**).

We next studied the regulating effects of Tregs on injury-associated SSCs, since Tregs have been observed communicating with tissue stem cells (24). We first observed the injury-induced expansion and distribution of SSCs within the injury site from Treg-depleted mice and control mice. Previously reported surface markers of skeletal stem and progenitor cells were used to distinguish SSC subsets (8, 9) and their differentiation within the injury site (Figure 2, A and B, and Supplemental Figure 4). Flow cytometry analysis of the callus tissue showed an immediate increased accumulation of SSCs, bone, cartilage, and stromal progenitor cells (BCSPs) and mature osteo-lineage (Thy1^+^) cells from day 7, which remained at high levels 1 month after bone injury (Figure 2B). These data indicated that SSCs accumulated in the callus immediately after injury and then were activated and gave rise to abundant skeletal progenitor cells and mature osteoblasts for bone repair. However, significant decreases in the percentages of total skeletal stem lineage cells, SSCs, BCSPs, and mature osteo-lineage cells were observed within the callus in the Treg-depletion group compared with the control group (Figure 2B). These results indicated that injury-induced SSC niche expansion was diminished under Treg-depletion conditions.

We also collected SSCs from callus tissues for bulk RNA-Seq analysis. In the control group, compared with SSCs from the uninjured bone tissues, SSCs from the injury sites displayed marked upregulation of genes related to stemness (e.g., *Ctsk*, *Sox9*, *Cd200*, and *Pdgfrb*) and osteogenic differentiation (e.g., *Alp*, *Ogn*, and *Runx2*). In the SSCs isolated from Treg-depleted mice, however, the expression of genes involved in stemness and osteogenesis was downregulated, while the expression of genes participating in apoptosis and senescence (e.g., *Casp4*, *Apaf1*, *Csf2ra*) was upregulated (Figure 2C). The volcano plots (Figure 2D) also showed that the osteogenic differentiation gene expression in SSCs was inhibited by Treg depletion. In addition, Gene Ontology (GO) term enrichment analysis of differentially expressed genes also confirmed that biological processes “stem cell differentiation,” “osteoblast differentiation,” and pathways “BMP signaling” and “TGF-β signaling” were more significantly enriched in SSCs from the injury site in control mice, while they were not enriched in Treg-depleted mice (Figure 2E).

We further isolated Tregs from mouse SPs and lymph nodes and cultured in vitro for 3 days, and then the supernatant was collected. From the same mice, we also isolated SSCs and subjected them to a standard osteogenic/adipogenic differentiation assay in the presence or absence of supernatant collected from cultured Tregs (Figure 2F). SSCs stimulated by the Treg supernatant showed significantly elevated osteogenic differentiation, revealed by increased alkaline phosphatase–positive (ALP-positive) staining colonies (Figure 2G). The oil red O staining revealed no significant difference in their adipogenic differentiation ability (Figure 2G).

Given the DT toxicity, we also constructed a bone-injury model in DT-treated WT mice. The results showed that DT itself didn’t affect the SSC accumulation, function, and bone healing after bone injury (Supplemental Figure 5). Together, these experiments demonstrated that the Treg depletion reduced SSC accumulation and impaired SSC osteo-lineage output during the injury-repair process.

### Identification of an “injury-responding” Treg subpopulation with bone-repair capacity.

**Figure 3:**
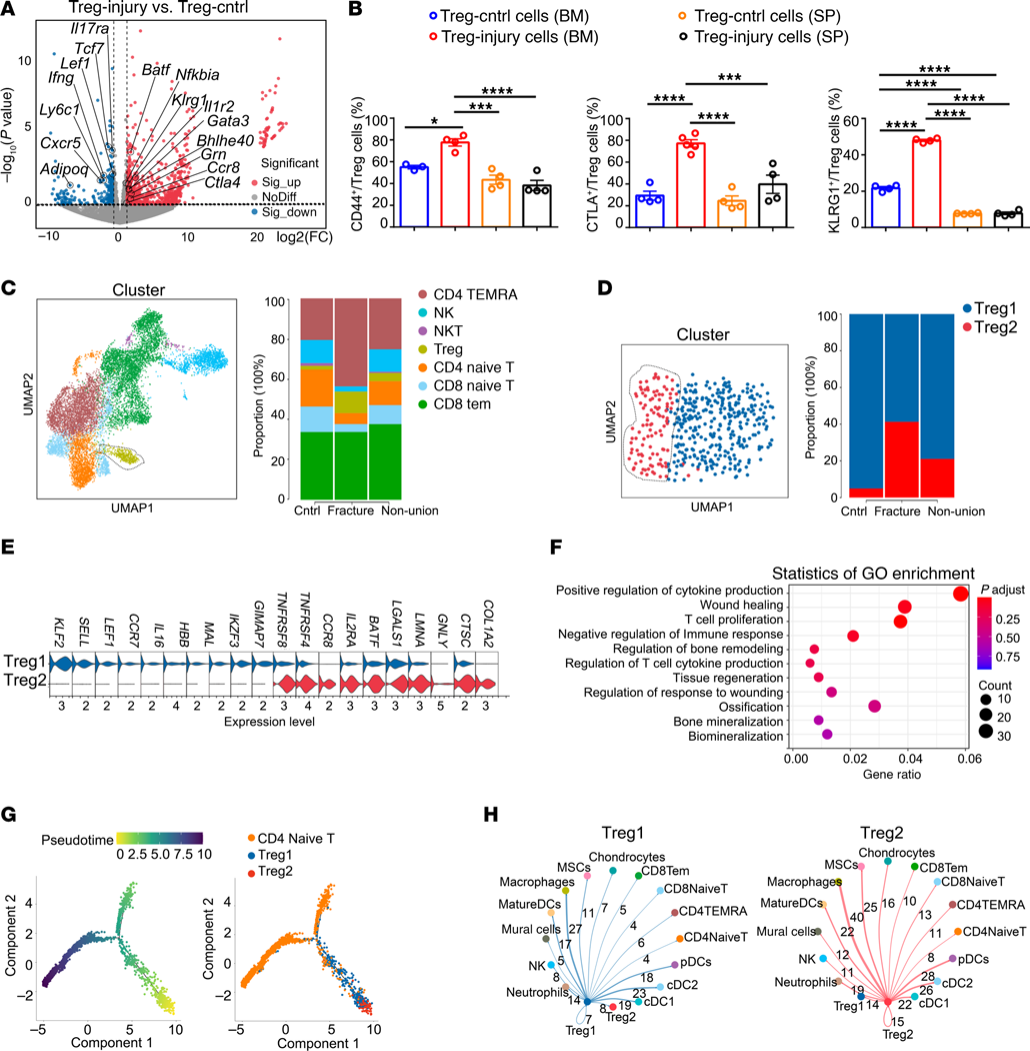
The distinct transcriptome of “injury-responding” Tregs within the injury site. (**A**) Volcano plots represent differentially expressed genes between Treg-Cntrl cells and Treg-injury cells. *n* = 3 per group. (**B**) The experiments compare the percentage of CD44-, CTLA-, and KLRG1-positive Tregs from the uninjured bone (blue), Tregs from the wound/callus tissue at the injury site (red), Tregs from the SP of uninjured mice (orange), and injured mice (gray). *n* = 3–5 per group. (**C**) UMAP plot showing clusters and cluster annotations of BM T and NK cell (left panel). Stacked bar graph showing percentages of cells in each cluster among total NK and T lymphocytes (right panel). (**D**) UMAP plot showing clusters and cluster annotations of BM Tregs (left panel) and stacked bar graph showing percentages of cells in each cluster among total Tregs (right panel). (**E**) Violin plot showing the top markers for Treg1 and Treg2 subpopulations. (**F**) GO enrichment of the biological process categories in Treg2 subpopulation compared with Treg1 subpopulation. (**G**) Pseudotime analyses showing the differentiation of Treg1 subpopulation and Treg2 subpopulation from CD4 naive T cells. (**H**) Network diagram of the interaction between Treg1 (right panel) or Treg 2 (left panel) cluster and other cells in the BM. The size of the circle represents the number of interactions with all other types of cells, and the thickness of the line represents the interaction number of cells between the line. All data are shown as mean ± SEM. *P ≤ 0.05; ***P ≤ 0.005; ****P ≤ 0.001, as determined by 1-way ANOVA with Bonferroni’s multiple-comparisons test.

Tregs have remarkable phenotypic plasticity and tissue specificity and can exhibit distinct features in response to a changing environment (33–35). Thus, we explored whether the bone-injury niche imparts distinct features beneficial for bone repair to these Tregs. Bull-RNA-Seq analysis showed upregulated transcripts in Tregs isolated from injured bone tissue (Treg-injury cells) encoded Treg activation markers (e.g., *Ctla4*, *Il1r2*, and *Tnf* receptor superfamily members) and effector molecules (e.g., *Gata3*, *Nfkbia*, and *Klrg1*). Conversely, Treg-injury cells made lower transcripts encoding proteins known to dampen the inflammation (e.g., *Ifng*, *Cxcr5*, and *Ill7a*) (Figure 3A). Flow cytometry analysis confirmed that significantly higher percentages of Treg-injury cells expressed functional markers (CD44, CTLA, and KLRG1) compared with the Treg-control cells and SP Tregs (Figure 3B). These data indicated that bone injury induced a significant phenotypic change of Tregs at the injury site.

Furthermore, we performed a bioinformatics analysis using a scRNA-Seq dataset of BM cells from a previous study analyzing immune cell constitution in intramedullary canal tissue obtained from human fracture patients (31). For the control group, the BM tissue was obtained when harvesting autologous bone graft. In the fracture group, BM samples were collected when patients underwent internal fixation of fracture. The BM sample for the nonunion group was collected at the time of surgical repair for femur fracture nonunion. Besides the decreased percentage of T cells in BM in the nonunion group (31), we found that the proportion of Tregs increased in fracture patients compared with control individuals and significantly decreased in nonunion patients (Figure 3C). Moreover, Tregs were clustered into 2 distinct populations, Treg1 and Treg2 subpopulations, as visualized by uniform manifold approximation and projection (UMAP) (Figure 3D). Interestingly, the Treg2 subpopulation was barely detected in control individuals and showed much higher proportion in the fracture group compared with the control group. And the frequency of the Treg2 subpopulation was much lower in the nonunion group compared with the fracture group (Figure 3D). In addition, the Treg2 subpopulation exhibited high expression of genes resembling effector Tregs (e.g., *IL2RA*, *TNFRSF4*, *TNFRSF18*) and preferentially expressed tissue Treg markers (e.g., *CCR8*, *BATF*, and *GNLY*), while the Treg1 subpopulation highly expressed transcripts (e.g., *SELL*, *TCF7*, and *LEF1*) indicative of resting phenotype (Figure 3E). GO term enrichment analysis revealed that the preponderance of biological processes related to the “regulation of stem cell differentiation,” “regulation of inflammatory response,” “ossification,” and “wound healing” in the Treg2 subpopulation (Figure 3F). Pseudotime analyses showed more terminated differentiation of the Treg2 subpopulation than the Treg1 subpopulation, originating from CD4 naive T cells (Figure 3G). Using CellPhoneDB2, a cell ligand/receptor pairing-based database (36), we identified a closer interaction and different pathway crosstalk between the Treg2 subpopulation and other cell populations in BM compared with that in the Treg1 subpopulation (Figure 3H and Supplemental Figure 6), among which, the CCR8-CCL1 pathway was only detected in the Treg2 cluster but not in theTreg1 cluster.

In summary, our data showed that bone injury induced the accumulation of a unique “injury-responding” Treg subpopulation (Treg2) at the injury site, which was enriched with reparative signatures related to the regulation of stem cell differentiation, secreted factors, cell-cell interactions, and immunoregulation, potentially involved in bone repair.

### Injury-responding Tregs are marked by CCR8 and are recruited to the injury site in a CCL1-dependent way.

**Figure 4:**
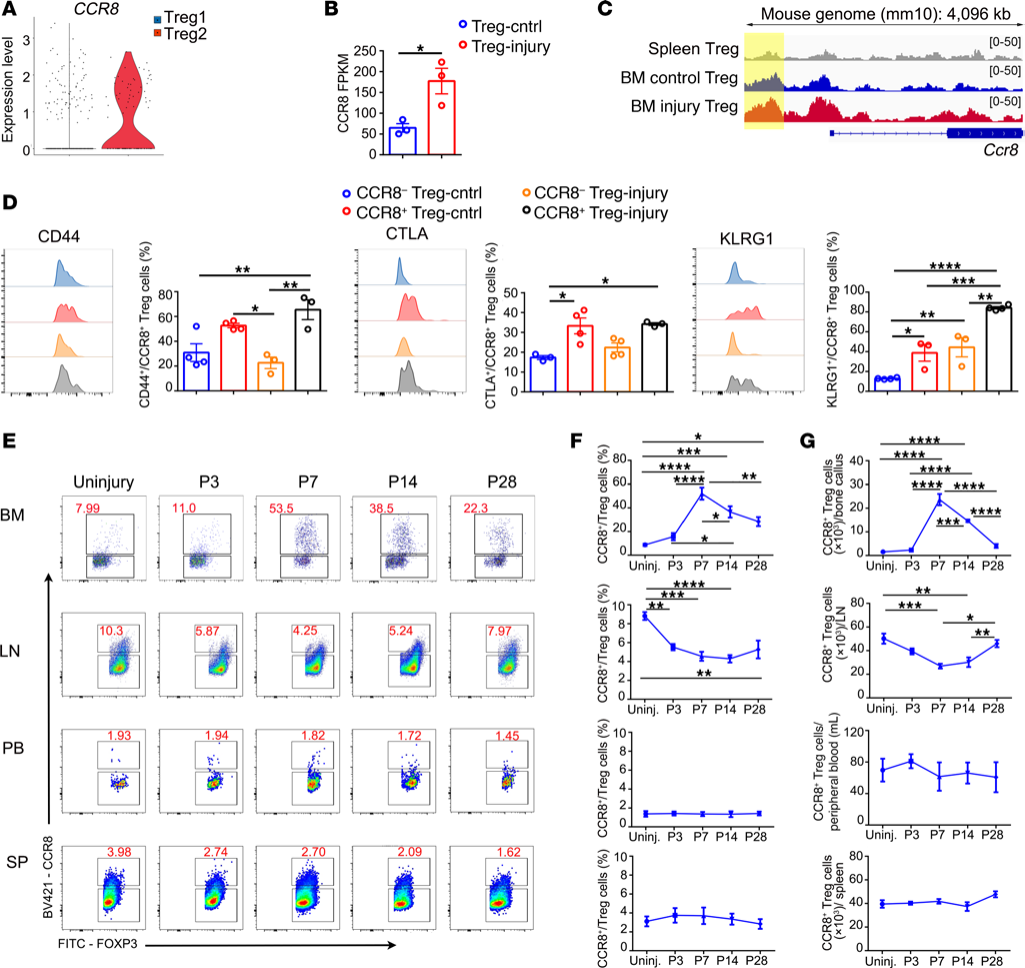
Bone injury–responding Treg population accumulates at the injury site marked by CCR8 and migrates from periphery. (**A**) Violin plots of *CCR8* gene expression in Treg1 subset and Treg2 subset. (**B**) Statistical analysis of *Ccr8* gene expression in Tregs from uninjured bone and injured bone. *n* = 3 per group. (**C**) Chromatin accessibility of the *Ccr8* locus in SP Tregs and BM Tregs in control and injury groups. (**D**) Representative histograms (left panels) and statistical analysis (right panels) of the expression of CD44, GTLA, and KLRG1 in CCR8^+/–^ Tregs from different sources: uninjured bone (blue for CCR8^–^ and red for CCR8^+^) and injury site (orange for CCR8^+^ and black for CCR8^–^). *n* = 3–4 per group. (**E**) Representative flow images of the proportions of CCR8^+^ Tregs among total Tregs in BM, adjacent inguinal LN, peripheral blood (PB), and SP before surgery and 3, 7,14, and 28 days after surgery. (**F**) Statistical analysis of the proportions of CCR8^+^ Tregs among total Tregs in BM, LN, PB, and SP at indicated time points. *n* = 4–5 per group. (**G**) The numbers of CCR8^+^ Tregs in BM, LN, PB, and SP at indicated time points. *n* = 4–5 per group. All data are shown as mean ± SEM. *P ≤ 0.05; **P ≤ 0.01; ***P ≤ 0.005; ****P ≤ 0.001, as determined by unpaired 2-tailed Student’s *t* test (**B**), 1-way ANOVA with Bonferroni’s multiple-comparisons test (**D**, **F**, and **G**).

Of note, our attention was quickly drawn to the preferentially expressed surface marker gene *CCR8* in the Treg2 cluster (Figure 4A), which has been identified as the most robustly and differentially expressed chemokine receptor in tissue Tregs (37–42). Bulk-RNA-Seq and ATAC-Seq data also confirmed higher mRNA expression level and more abundant chromatin accessibility of *Ccr8* in Tregs cells isolated from injury bone tissue (Figure 4, B and C). Compared with CCR8^–^ Tregs, CCR8^+^ Tregs expressed much higher levels of activation markers, CD44, CTLA, and KLRG1. And the KLRG1^+^CCR8^+^ Treg proportion at the injury site was much higher than in control BM tissue (Figure 4D). Moreover, consistent with the rising tendency of total Tregs at the injury site, the fraction of CCR8^+^ Tregs elevated from day 3 peaked on day 7 (51.9% ± 11.28% versus 8.3% ± 2.04% on day 0) and remained high until at least 28 days after injury (Figure 4, E and F). All these data suggested that the CCR8 marked the unique bone-injury–responding Tregs that accumulated at the injury site.

**Figure 5:**
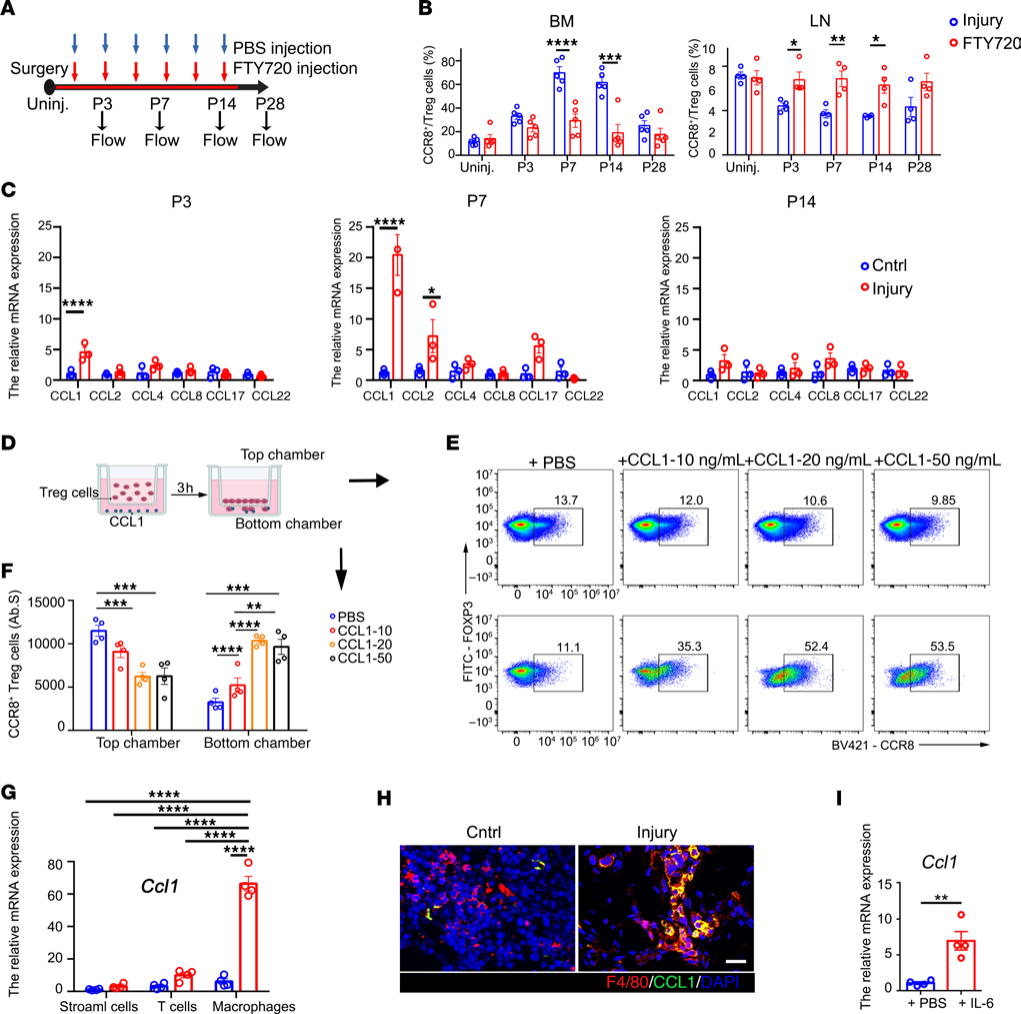
Macrophage-derived CCL1-mediated CCR8^+^ Tregs migration into the injury site. (**A**) Schematic diagram showing the FTY720 treatment protocol. (**B**) The fractions of CCR8^+^ Tregs among total Tregs in the control group (Cntrl) and FTY720-treated group in BM and adjacent lymph nodes at indicated time points. *n* = 4–5 per group. (**C**) Transcript levels of chemokine ligand gene transcripts in the wound/callus tissue and control tissue were quantified by qRT-PCR before surgery and on days 3, 7, and 14 after injury. *n* = 3 per group. (**D**) Schematic diagram showing CCL1-mediated chemotaxis assay protocol. (**E**) Representative flow images of the proportions of CCR8^+^ Tregs among total Tregs in top/bottom chambers treated with CCL1 at indicated concentrations. (**F**) The number of CCR8^+^ Tregs in top/bottom chambers treated with CCL1 at indicated concentrations. *n* = 4 per group. (**G**) The relative expression levels of *Ccl1* in stromal cells, T cells, and macrophages derived from BM tissue in control and bone injury mice. *n* = 4 per group. (**H**) Representative images of colocalization of F4/80 (red) and CCL1 (green) in control/injured bone tissue. Scale bars: 10 μm. (**I**) The relative expression level of *Ccl1* in BM macrophages treated with PBS or IL-6. *n* = 4 per group. All data are shown as mean ± SEM. *P ≤ 0.05; **P ≤ 0.01; ***P ≤ 0.005; ****P ≤ 0.001, as determined by 2-way ANOVA with Bonferroni’s multiple-comparisons test (**B**, **C**, and **F**), 1-way ANOVA with Bonferroni’s multiple-comparisons test (**G**), or unpaired 2-tailed Student’s *t* test (**I**).

Next, we asked the origin of the accumulated CCR8^+^ Tregs at the injury site. In contrast to the increased proportion and number of CCR8^+^ Tregs at the injury site, CCR8^+^ Treg proportion and number in the adjacent inguinal lymph node was decreased after surgery (Figure 4, E–G). In the SP and peripheral blood, the proportion and the number of CCR8^+^ Tregs remained unchanged (Figure 4, E–G). These data suggested the migration of CCR8^+^ Tregs from periphery to the injury site. Treatment with FTY720, an S1P receptor antagonist that inhibits lymphocyte peripheral tissue immigration (26), significantly reduced the percentage of CCR8^+^ Tregs at the injury site, which confirmed that peripheral CCR8^+^ Tregs contributed to the accumulation of CCR8^+^ Tregs at the injury site (Figure 5, A and B).

We further investigated the mediators inducing the CCR8+ Treg migration after injury through utilizing a quantitative reverse transcription PCR–based (qRT-PCR based) chemokine-expression array to examine the expression level of known CCR8 ligands. We observed a pronounced and highly preferential increased expression of Ccl1 within the wound/callus tissue, which was detectable on day 3 and sustained to day 14 after injury (Figure 5C). In vitro chemotaxis assay demonstrated the effect of CCL1 in recruiting CCR8+ Tregs (Figure 5, D–F). Furthermore, we showed that macrophages were a major source of CCL1 in BM (Figure 5G). Immunofluorescent (IF) staining showed that CCL1 was expressed at a much higher level in macrophages at the injury site (Figure 5H). IL-6, which has been reported highly expressed in the early post–bone injury period (43, 44), significantly increased Ccl1 gene expression in macrophages (Figure 5I). These data suggested that local production of CCL1 from macrophages was related to the early acute inflammation following bone injury.

**Figure 6:**
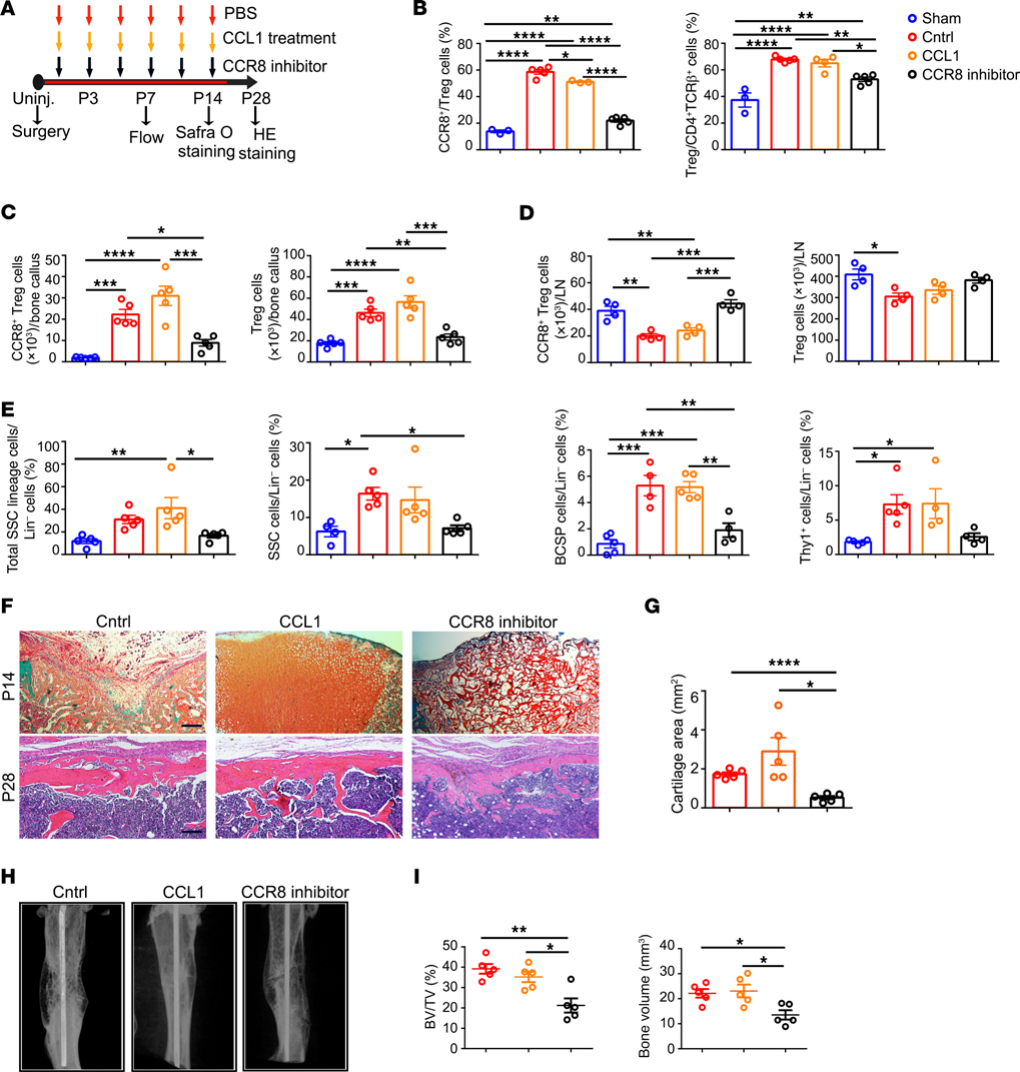
CCR8^+^ Tregs promote bone repair in a CCL1-dependent manner. (**A**) Schematic diagram showing the CCL1 and CCR8 inhibitor treatment protocol. (**B**) Proportions of CCR8^+^ Tregs among Tregs and Tregs among CD4^+^ TCRβ^+^ cells from uninjured group (sham, blue) and from injury site at control group (Cntrl, red), CCL1-treated group (CCL1, yellow), and CCR8 inhibitor–treated group (black) on 7 days after surgery. *n* = 3–5 per group. (**C**) The numbers of CCR8^+^ Tregs (left) and total Tregs (right) in bone callus tissue on P7. *n* = 5 per group. (**D**) The numbers of CCR8^+^ Tregs (left) and total Tregs (right) in adjacent lymph nodes on P7. *n* = 4 per group. (**E**) Frequencies of total SSC lineage cells, SSCs, BCSPs, and Thy1^+^ cells in total Lin^–^ cells at the injury site on P7. *n* = 4–5 per group. (**F**) Safranin O staining (upper) and H&E staining (lower) of bone tissues at injury site in Cntrl, CCL1-treated group, and CCR8 inhibitor–treated group on P14 and P28, respectively. Scale bars: 200 μm. (**G**) The Safranin O images were analyzed using ImageJ, version 1.48. *n* = 5 per group. (**H**) Representative μCT images showing fracture healing. (**I**) Quantitative analysis of bone volume fraction (BV/TV) and bone volume of callus tissue. *n* = 5 per group. All data are presented as mean ± SEM. *P ≤ 0.05; **P ≤ 0.01; ***P ≤ 0.005; ****P ≤ 0.001, as determined by 1-way ANOVA with Bonferroni’s multiple-comparisons test.

Then a series of loss- and gain-of-function experiments were performed to evaluate the function of the CCR8/CCL1 axis in the accumulation of CCR8^+^ Tregs after injury (Figure 6A). CCR8 inhibitor ML604086, demonstrating a significant effect in inhibiting CCL1 binding to CCR8 (Supplemental Figure 7), and CCL1-neutralizing antibody (CCL1 inhibitor) were employed to interrupt the CCL1/CCR8 axis (Figure 6A and Supplemental Figure 8A).

Within the injury site, the proportions and numbers of CCR8^+^ Tregs and total Tregs were significantly reduced in mice treated with CCR8 inhibitor or CCL1 inhibitor compared with untreated mice on day 7 after surgery (Figure 6, B and C, and Supplemental Figure 8, B and C). The CCR8^+^ Treg proportions and numbers in the adjacent lymph node were increased in the CCR8 inhibitor –and CCL1 inhibitor–treated groups (Figure 6D and Supplemental Figure 8D). These results showed that CCR8^+^ Tregs expanded during the bone-repair response in a CCL1-dependent manner.

Since CCR8 inhibitor and CCL1 inhibitor treatment impaired CCR8^+^ Treg accumulation at the injury site, we further explored whether bone healing was affected. We first examined the distribution of SSC lineage cells at the injury site on day 7 after injury. The results showed that the total SSC lineage cell, SSC, and BCSP frequencies significantly declined in the CCR8 inhibitor/CCL1 inhibitor-treated groups (Figure 6E and Supplemental Figure 8E). Safranin O staining results indicated that the CCR8 inhibitor/CCL1 inhibitor treatment decreased cartilage formation on P14 (Figure 6, F and G, and Supplemental Figure 8F). H&E staining confirmed a significant impairment of defect healing in the CCR8 inhibitor/CCL1 inhibitor-treated groups on P28 (Figure 6F and Supplemental Figure 8G). Fracture model also showed prohibited bone repair in CCR8 inhibitor/CCL1 inhibitor–treated groups (Figure 6, H and I, and Supplemental Figure 8, H and I). Together, these findings demonstrated that CCR8^+^ Tregs enabled bone repair in a CCL1-dependent manner.

### Bone-injury–responding Treg-derived PGRN promotes SSC osteogenic function and bone repair.

**Figure 7:**
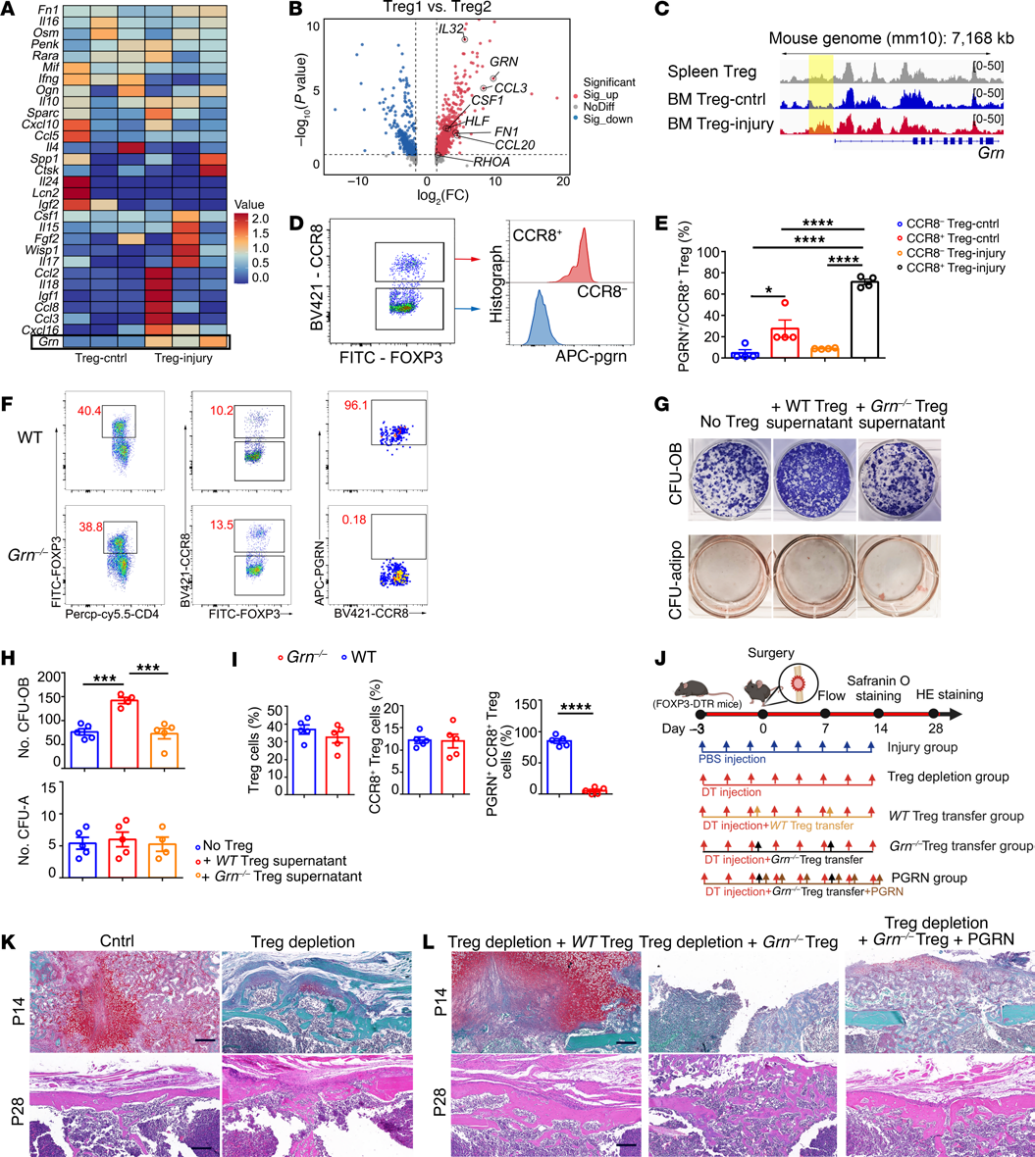
Bone injury–responding Tregs display bone repair function dependent on secretion of PGRN. (**A**) Heatmap showing the expression of secreted factors in Tregs at injury site and Tregs in the control BM. *n* = 3 per group. (**B**) Volcano plot representation of a comparison of secreted gene-expression profiles between Treg1 subset and Treg2 subset. (**C**) Chromatin accessibility of the *Grn* locus in SP Tregs and BM Tregs in control and injury group. (**D**) Representative images of the expression of PGRN in CCR8^+^ Tregs and CCR8^–^ Tregs. (**E**) Quantification of the expression of PGRN in CCR8^+^ Tregs and CCR8^–^ Tregs. *n* = 4 per group. (**F**) Representative images showing the percentage of total Tregs, CCR8^+^ Tregs, and PGRN^+^CCR8^+^ Tregs in WT mice and *Grn^–/–^* mice. (**G**) Representative images of colonies of osteogenic (CFU-OB) and adipogenic (CFU-Adipo) assays showing the effect on the control with no Treg conditioned medium, with WT Treg conditioned medium, and with *Grn^–/–^* Treg conditioned medium. (**H**) Quantification of CFU-OB and CFU-Adipo colonies. *n* = 4–5 per group. (**I**) Statistical analysis of the proportions of Tregs in CD4^+^TCRβ^+^ cells, the proportion of CCR8^+^ Tregs among total Tregs, and the percentage of PGRN^+^CCR8^+^ Tregs in CCR8^+^ Tregs in WT mice and *Grn^–/–^* mice. *n* = 5 per group. (**J**) Diagram of the experimental protocol showing the transfer of WT/*Grn^–/–^* Tregs into Treg-depletion mice after injury. (**K** and **L**) Safranin O staining of bone tissues on day 14 after injury operation (upper). H&E of bone tissues on day 28 after injury operation (lower). Scale bars: 200 μm. All data are shown as mean ± SEM. *P ≤ 0.05; ***P ≤ 0.005; ****P ≤ 0.001, as determined by 1-way ANOVA with Bonferroni’s multiple-comparisons test (**E** and **H**) or unpaired 2-tailed Student’s *t* test (**I**).

In search of the underlying mechanism involved in the bone-repair role of this injury-responding Treg subpopulation, we focused on their expression of secreted factors. Several genes encoding classical trophic factors (25–30), including *Areg*, *Penk*, *Spp1*, and *Sparc*, known to promote tissue repair, did not show a significant difference between Treg-injury cells and Tregs from the uninjured bone in our data (Figure 7A). However, genes encoding an osteogenic factor (45, 46), PGRN, were preferentially expressed in Treg-injury cells (Figure 7A) and the Treg2 subpopulation (Figure 7B). ATAC-Seq analysis confirmed more abundant chromatin accessibility of *Grn* in Tregs at the injury site (Figure 7C). In addition, flow cytometry analysis showed that CCR8^+^ Tregs expressed much higher levels of PGRN compared with CCR8^–^ Tregs and the expression level was further induced by injury (Figure 7, D and E).

These results prompted us to explore the role of Treg-derived PGRN in mediating SSC function and bone repair through using *Grn^–/–^* mice. *Grn* deletion did not affect the percentage of total Tregs and CCR8^+^ Tregs (Figure 7, F and I). *Grn^–/–^* Tregs showed similar immunosuppressive function compared with WT Tregs (Supplemental Figure 9, A and B). In vitro multipotential differentiation assay showed that supernatant from cultured *Grn^–/–^* Tregs displayed an impaired effect in inducing SSC osteogenic differentiation compared with the SSCs treated with WT Treg supernatant (Figure 7, G and H). Furthermore, we adoptively transferred Tregs derived from WT or *Grn^–/–^* mice into Treg-depleted mice after bone injury (Figure 7J). The results showed that increased IFN^+^ and TNF-α^+^ Teff cell percentages induced by Treg depletion were decreased by transferring *Grn^–/–^* Tregs or WT Tregs (Supplemental Figure 9, C and D). However, the *Grn^–/–^* Treg transferring group still showed massive fibrous tissue infiltration at the injury site without cortical fusion (Figure 7, K and L, and Supplemental Figure 10), while transferring of WT Tregs or adding of PGRN protein resulted in better healing outcomes with both callus formation and cortical fusion (Figure 7, K and L, and Supplemental Figure 10). These in vivo and in vitro data collectively demonstrated the importance of PGRN in mediating Treg-SSC crosstalk and thereby promoting bone repair.

### BATF drives PGRN expression in the injury-responding Tregs.

**Figure 8:**
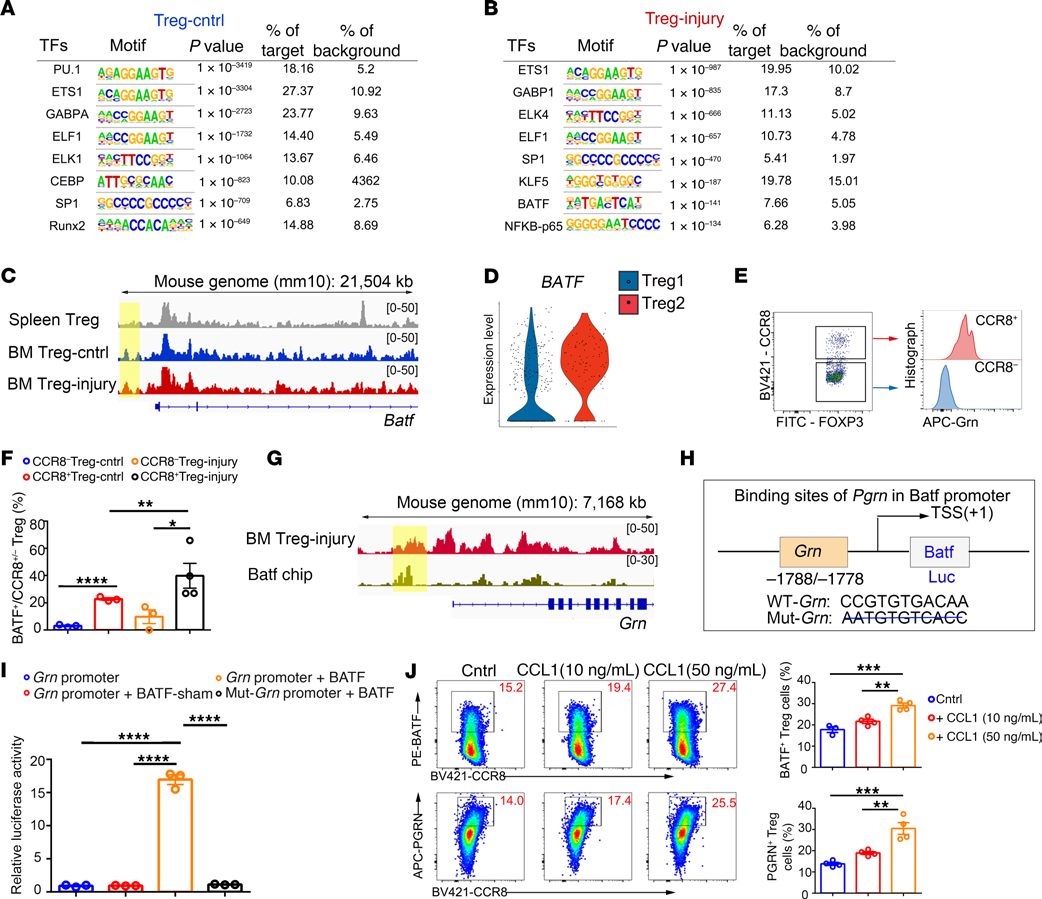
BATF modulates PGRN expression in the injury-responding Tregs. (**A**) De novo motif-enrichment analysis of ATAC-Seq peaks associated with Treg-Cntrl cell–expressed genes encoding transcription factors. Random background regions serve as a control. (**B**) De novo motif-enrichment analysis of ATAC-Seq peaks associated with Treg-injury cells–expressed genes encoding transcription factors. Random background regions serve as a control. (**C**) Chromatin accessibility of the *Batf* locus in SP Tregs and BM Tregs in control and injury group. (**D**) Violin plot showing the expression level of *BATF* in Treg1 subset and Treg2 subset. (**E**) Representative images of the expression of BATF in CCR8^+^ Tregs and CCR8^–^ Tregs. (**F**) Quantification of the expression of BATF in CCR8^+^ Tregs and CCR8^–^ Tregs. *n* = 3 per group. (**G**) Chromatin accessibility of the *Grn* locus in BM Tregs with BATF ChIP-Seq data. (**H**) Diagram graph showing the binding sites of *Grn* with *Batf*. (**I**) Dual luciferase reporter assay in HEK293A cells cotransduced with luciferase reporter driven by WT or mutant *Grn* promoter and expression plasmid of *Batf*. *n* = 3 per group. (**J**) Representative images and quantification of BATF^+^ and PGRN^+^ CCR8^+^ Tregs with or without treatment with CCL1 (10 ng/ml or 50 ng/ml). *n* = 3–4 per group. All data are shown as mean ± SEM. *P ≤ 0.05; **P ≤ 0.01; ***P ≤ 0.005; ****P ≤ 0.001, as determined by 1-way ANOVA with Bonferroni’s multiple-comparisons test.

We next explored the driving transcription factors that are essential for repair capacity of injury-responding Tregs in the bone-injury tissue. De novo motif analysis of DNA sequences enriched under the ATAC-Seq peaks showed the top significantly enriched transcription factor binding sites for genes expressed in Treg-control cells and Treg-injury cells, and among which, transcription factor binding sites for *Batf* were more highly enriched in Treg-injury cells (Figure 8, A–C). scRNA-Seq and bulk-RNA-Seq also showed that the gene encoding BATF was highly expressed in the Treg2 cluster and Treg-injury cells (Figure 3A and Figure 8D). Flow cytometry analysis also confirmed that CCR8^+^ Tregs expressed much higher levels of BATF compared with CCR8^–^ Tregs (Figure 8, E and F).

Since BATF has been widely shown to reorganize chromatin structure and differentiation of T cells (47), we asked whether BATF is crucial for injury-responding Treg repair capacity, especially for their secretion of PGRN. We first overlaid the Batf chip seq data (NCBI’s Gene Expression Omnibus GSE39756) with *Grn* chromatin accessibility through ATAC-Seq data and found that the *Grn*-specific binding peak on chromosome 11 maps the 5′-region of the *Batf* gene (Figure 8G). Therefore, we speculated that BATF may bind to the *Grn* promoter to activate its transcription. The JASPAR database identified the predicted *Grn*-binding sites within the BATF (Figure 8H). The binding sites with the highest score were located in –1788 to –1778 bp, upstream of the transcription start site.

To further determine the effect of BATF-mediated *Grn* promoter activation, we mutated the BATF-binding sites in the *Grn* promoter and individually transfected the respective plasmids containing either mutated or WT promoter regions into HEK293A cells overexpressing BATF. As expected, we found significantly increased *Grn* promoter activity induced by BATF compared with the activity of the mutated *Grn* promoter (Figure 8I), which indicated that the BATF highly promoted the transcription of *Grn*.

We then examined whether CCL1 increased the BATF expression level of Tregs when mediating the accumulation of Tregs to the injury site. To test this, we cultured isolated Tregs in vitro with or without treatment of CCL1. Flow cytometry results showed that CCL1 treatment increased the positive percentage of BATF and also PGRN in Tregs (Figure 8J), which suggested that CCL1 could be a potential therapeutic strategy for improving bone-repair capacity of Tregs and bone-related disease.

## Discussion

Upon bone injury, numerous immune cells and stromal cells accumulate within the injury site to constitute a specific local microenvironment for recruiting and activating endogenous stem cells and initiating the repair process (11). Advanced knowledge of the crucial signals that instruct SSC function at the injury site is necessary for guiding bone repair and regeneration. Macrophages and neutrophils have been widely studied for their important roles in mediating stem cell recruitment and bone repair. Still, their accumulation is mainly in the early inflammation phase, not in the middle or late stage of the repair process (5, 12). Additional factors are necessary for switching off the inflammatory phase of wound healing and initiating the repair/regeneration process. The present study shows the indispensable and long-lasting role of Tregs on bone repair through direct interaction with SSCs and not simply through controlling inflammation. Finally, we observed a substantial increase of the number of Tregs in the injury niche through the whole repair process, which peaked on day 7, then remained at a high level, and slowly returned to a normal level 28 days after injury. Second, Treg depletion led to decreased SSC number at the injury site by inhibiting SSC accumulation and osteogenic differentiation, thereby generating an undersized, improperly composed callus after bone injury.

Tregs display remarkable tissue specificity and can exhibit distinct features in response to external stimulation (34, 35). Thus, it is essential to examine whether Tregs within the bone-injury site differ from those outside the injury site in phenotype, function, and transcriptional profile, as these may help explain the mechanisms that instruct Tregs to communicate with SSCs. Here, combined with scRNA-Seq of BM Tregs in fracture patients and bulk-RNA-Seq of Tregs isolated from mouse injured femur tissue, we found that a bone-injury–responding Treg population marked by CCR8 displayed a distinct tissue-repair phenotype. Compared with the CCR8^–^ Tregs, CCR8^+^ Tregs showed a higher activation level and tissue-repair capacity by increased expression of CD44, CTLA KLRG1, and BATF and enrichment with genes related to the bone formation pathway and stem cell regulation pathway. Interestingly, the observed phenotype presented 2 sets of features: one associated with stem cell regulation that has been previously reported in multiple organs and tissues and another characteristic for bone.

It is worth noting that CCR8^+^ Tregs also exist in the bone tissue, lymph nodes, and SP but in a very low proportion under normal conditions. The CCR8^+^ Treg number significantly increased at the injury site along with decreased number of CCR8^+^ Tregs in the adjacent lymph node. FTY720 treatment inhibited this change, which suggested the migration of CCR8^+^ Tregs from periphery to the injury site. Upon injury, the CCR8 ligand CCL1 was highly produced from macrophages within the injury site, a response triggered by the local inflammatory cytokines. In vivo evidence has been procured through treatment with CCR8 inhibitor/CCL1 inhibitor, complimented by in vitro evidence via a chemotaxis assay, affirming the capacity of CCL1 to mobilize CCR8^+^ Tregs to the injury site, which indicated the pivotal role CCL1 plays in bone repair.

More importantly, these injury-associated CCR8^+^ Tregs secreted specific osteogenic factor PGRN to mediate their crosstalk with SSCs. Previously reported classical tissue repair factors such as AREG, PENK, or SPP1 did not show significant increased expression in these CCR8^+^ Tregs. The loss of function of PGRN didn’t affect the immunosuppressive function in Tregs, but impaired their role in regulating SSCs’ osteogenesis function and bone healing. Our data suggested that PGRN could be a potential therapeutic target to treat bone diseases in which the patients have a lower proportion of Tregs, such as AIDS-related fractures and osteoporosis. More importantly, a transcription factor, BATF, has been demonstrated to regulate the secretion level of PGRN in the injury-responding Tregs.

We identified a specific bone-repair Treg subpopulation highly expressed surface marker CCR8, which infiltrated into the bone-injury site in response to CCL1 and directly cross-talked with SCCs through secreting PGRN to promote SSC osteogenic function and the new bone formation. Targeting CCR8^+^ Tregs and their products could provide a potential strategy for accelerating bone healing and treating skeletal diseases.

## Methods

### Sex as a biological variable.

Our study exclusively examined male mice to reduce female sexual cycle–related variation.

### Mouse strain.

All mice were maintained on a C57BL/6j background. The B6.129(Cg)-*Foxp3*^tm3Ayr^/J (*Foxp3*-DTR mice) (48) from Jackson Laboratory were used for the pharmacological ablation of Tregs. The human DT receptor expressing *Foxp3*-DTR mice was generated by integrating human DTR and EGFP sequences into the stop codon of the *Foxp3* gene. *Grn*^–/–^ mice from Cyagen Biosciences (C57BL/6J-Grnem1C/Cya, S-KO-02346) were used for isolating *Grn*-deleted Tregs. Mouse genotypes were determined by PCR of genomic DNAs extracted from mouse tails. Primer sequences are listed in Supplemental Table 2. Two-month-old male mice were used and analyzed in all experiments. Control male littermates were analyzed in all experiments. All mice were housed in specific pathogen–free animal care facilities under a 12-hour light/12-hour dark cycle with a temperature of 18–24°C and humidity of 35%–60%.

Treg ablation in *Foxp3*-DTR mice was induced by DT (5 μg/kg, catalog D0564, Sigma-Aldrich) immediately after defect surgery or sham surgery and repeated every 2 days until sacrifice. To retain lymphocytes in secondary lymphoid tissue, FTY720 (catalog SML0700, Sigma-Aldrich) was injected intraperitoneally into mice at 1 mg/kg, 1 day before surgery and repeated every 2 days until sacrifice.

### Bone defect injury models.

A unilateral midshaft femoral defect injury model was produced in the left femur as previously described (49). Mice were anesthetized with isoflurane gas, and a small incision was made medial to the femur tuberosity. Standardized femur injury was performed 5 mm distal to the growth plate using a Micro-Drill with a 2 mm diameter stainless steel burr, and wounds were sutured. After recovery, animals were returned to housing cages. All animals received buprenorphine pre- and postoperatively for pain control.

Modified Bonnarens and Einhorn’s adapted fracture method was used for the fracture study (50). Isoflurane gas was used to anesthetize mice, and buprenorphine was given to all experimental animals before surgery. After making an anterior longitudinal midline incision centered over the knee joint, a 30-gauge needle, as an intramedullary pin, was driven through the distal femur toward the femoral head after subluxation of the patella. The needle was clipped and gently nested into the distal femur to avoid soft tissue damage. A custom guillotine-style fracture device, driven by a dropped weight, was used to generate femoral fracture. The weight was determined as the amount needed, from a defined height, to generate an impact leading to a focal transverse fracture at the mid-diaphysis.

### Histology and immunohistochemistry.

Femurs were collected at the indicated time points following surgery. Specimens for paraffin sections were fixed for 1 day in 4% paraformaldehyde in PBS at 4°C and then decalcified in 15% EDTA (catalog E1170, Solarbio). Decalcified femoral sections were stained with H&E or Safranin O according to the manufacturer’s protocol. TRAP activities in femur bones were tested in control mice and Treg-depleted mice, respectively, according to the manufacturer’s protocol. All bright-field images were captured on an Olympus BX-51 upright light microscope with an Olympus DP70 camera.

### Immunofluorescence staining.

Femur tissue was quickly harvested and embedded in OCT compound (catalog 4583, Sakura). Then, immunofluorescent staining was performed on 7 μm cryosections. Sections were blocked with 1% BSA for 60 minutes, permeabilized with 0.1% Triton X-100 in PBS for 15 minutes, and then incubated with primary antibodies overnight at 4°C. Later, the tissue sections were incubated with the appropriate fluorescent secondary antibodies for 1 hour at room temperature, avoiding exposure to light. Subsequently, DAPI was used to label the nuclei. Confocal microscopy (FluoView FV1000; Olympus) was used to capture images.

### Micro-computed tomography analysis.

Micro-computed tomography (μCT) scanning was performed to measure the microstructure of the bone callus. The femurs were dissected with the muscle left intact (to avoid bone callus damage), immediately fixed overnight in 4% paraformaldehyde at 4°C, and stored in 70% ethanol, until scanned by μCT (Scanco μCT-100) with an isotropic voxel size of 12 μm. μCT analysis was performed using CTAn (Bruker) in accordance with the recommendations of the American Society for Bone and Mineral Research (51). Volumes of interest (VOI) was defined by manually outlining a region of interest encompassing the callus. All analyses were performed in a blinded fashion.

### Immune cell isolation and flow cytometry.

To obtain cells from the injury site, the injured femur was harvested from the hip joint to the knee joint to avoid disturbing the callus tissue. Most of the surrounding soft tissues were removed with care so as not to disturb the injury tissue. The top and bottom portions of the femur at approximately 2 mm above and below the injury area were excised to obtain the bone callus (including the nearby BM). The remaining tissue was cut into pieces and then transferred to a dish with 1 mL of digestion buffer containing 1 mg/ml of collagenase D (catalog 11088882001, Roche), 2 mg/ml of dispase II (catalog 10165859001, Roche), and 10,000 unit/ml of DNase I (catalog 4716728001, Roche). Callus tissue was digested for 10 minutes at 37°C under gentle agitation and filtered through 70 μm nylon mesh to acquire single-cell suspension. Splenocytes and inguinal lymph node from the operated side were dissociated into a single-cell suspension by mechanical disruption and filtering through 70 μm nylon mesh. Venous blood was sampled in 10.0 ml Becton Dickinson (BD) Vacutainer blood collection tubes containing ethylenediamintetraacetic acid. For all samples, erythrocytes were removed with red blood cell lysis buffer (catalog 420302, BioLegend).

For the analysis of cell-surface markers, single cells were stained with indicated antibodies in PBS containing 2% FBS (catalog 10-082-147, Gibco, Thermo Fisher Scientific) at 4°C in the dark. FOXP3 and other intracellular markers were stained with corresponding antibodies using the FOXP3/Transcription Factor Staining Buffer Set (catalog 00-5523-00, eBioscience) according to the manufacturer’s instructions. For PGRN staining, cells were stained with anti-PGRN and rabbit IgG isotype control for PGRN, respectively, followed by the incubation of APC-labeled anti-rabbit secondary antibody. To determine cytokine expression, cells were stimulated with phorbol12-myristate 13-acetate (50 ng/ml, catalog P1585-1MG, Sigma-Aldrich), ionomycin (1 μM, catalog I3909-1ML, Sigma-Aldrich), Golgi Stop (catalog 554724, BD Pharmingen), and Golgi Plug (catalog 555029, BD Pharmingen) for 4 hours. At the end of stimulation, cells were stained with fixable viability dye eFluor 780 and the indicated antibodies for cytokines listed in Supplemental Table 2 using the Intracellular Fixation and Permeabilization Buffer Set (catalog 88-8824-00, eBioscience) following the manufacturer’s instructions.

All staining was performed on ice to maintain cell morphology. Flow cytometric data were acquired using a BD FACSdiva LSR-II flow cytometer (BD Biosciences) and analyzed using FlowJo software, version 10.0.6. (FlowJo LLC). Gating determinations were performed using fluorescence minus one (FMO) and isotype controls.

### SSC isolation and flow cytometry.

Callus tissue and control limbs were dissociated by mechanical and enzymatic digestion using 1 mg/ml of Collagenase P (catalog 11213857001, Roche), 2 mg/ml of dispase II, and 10,000 units/ml of DNase I for 1 hour at 37°C under gentle agitation. After digestion, cells were passed through a 40 μm cell strainer and washed with staining buffer (PBS containing 2% FBS). The digested cells were subsequently blocked with BD Pharmingen purified rat anti-mouse CD16/CD32 antibody for 10 minutes on ice and then stained with indicated antibodies in the dark for 1 hour on ice. Cells were then washed several times and resuspended in a staining buffer with DAPI (1 μg/ml, catalog D1306, Invitrogen). Primer sequences are listed in Supplemental Table 1. The strategy to sort skeletal progenitors is diagrammed in Supplemental Figure 4. Therefore, the total SSC lineage cells (CD45^−^Ter119^−^CD31^−^Thy1^−^6C3^−^CD51^+^), the SSC cells (CD45^−^Ter119^−^CD31^−^Thy1^−^6C3^−^CD51^+^CD105^−^), the BCSP cells (CD45^−^Ter119^−^CD31^−^Thy1^−^6C3^−^CD51^+^CD105^+^), and the Thy1^+^ cells (CD45^−^Ter119^−^CD31^−^CD51^+^Thy1^+^6C3^−^) were collected from the callus tissues or control limbs.

### Antibodies.

All antibodies used in this study are listed in Supplemental Table 2.

### Generation of Tregs in vitro culture supernatant.

Murine CD4^+^TCRβ^+^CD25^hi^GFP^+^ Tregs were sorted from SP and lymphoid tissue and stimulated with anti-CD3/CD28 DynaBeads (1 cell to1 bead, catalog 11453D, Invitrogen) in the presence of mIL-2 (50 U/ml, catalog 402-ML, R&D) for 24 hours. Anti-CD3/CD28 DynaBeads were further removed from the culture. Mouse Tregs were cultured in RPMI 1640 medium (plus β-mercaptoethanol, catalog M7522, Sigma-Aldrich) supplemented with 10% FBS, 1% GlutaMax (catalog 11360070, Gibco), 1% sodium pyruvate (catalog 11360070, Gibco, Thermo Fisher Scientific) and 1% penicillin/streptomycin (catalog 1507006, Gibco, Thermo Fisher Scientific). The culture medium was changed every 2 to 3 days, followed by the resupplementation of cytokines. After 4 days, the supernatant was collected and stored at –80°C.

To investigate the function of PGRN, CD4^+^TCRβ^+^CD25^hi^ Tregs were isolated from *Grn*^–/–^ mice and cultured in vitro as previously described. After 4 days, the supernatants were collected and stored at –80 °C.

### SSC culture with Treg culture supernatant and multiple differentiation assay.

Sorted SSCs were cultured in MEM-α medium (catalog 12561056, Gibco, Thermo Fisher Scientific) containing 10% FBS and 1% penicillin/streptomycin for 1 week. Then SSCs at passage 1 were plated in a single well of a 6-well plate at the density of 1 × 10^4^ cells/ml for 12 hours. To induce osteoblast differentiation, cells were cultured with a complete culture medium supplemented with 1 μM dexamethasone (catalog D4902, Sigma-Aldrich), 10 mM β-glycerophosphate (catalog G9422, Sigma-Aldrich), and 50 μg/ml ascorbic acid (catalog A5960, Sigma-Aldrich) for 14 days. Treg culture supernatant was diluted to 1:5 and added to SSC culture medium. The culture medium was replaced every 3 days for up to 2 weeks. Cells were then fixed with 4% paraformaldehyde for 15 minutes and stained with the BCIP/NBT ALP Staining Kit (catalog C3206, Beyotime) following the manufacturer’s instructions. To induce adipocyte differentiation, cells were cultured with a full culture medium containing 1 μM dexamethasone (catalog D4902, Sigma-Aldrich), 0.5 mM IBMX (3-Isobutyl-1-methylxanthine, catalog I5879, Sigma-Aldrich) and 1 μg/ml insulin (catalog I6634, Sigma-Aldrich). Culture media were exchanged with fresh media every 3–4 days for up to 2 weeks. Treg culture supernatant was diluted to 1:5 and added to SSC culture medium. Cells were then fixed with 4% paraformaldehyde for 15 minutes and stained with 0.3% oil red O solution (catalog O0625, Sigma-Aldrich).

### RNA preparation, qRT-PCR, and bulk RNA-Seq.

Total RNA from the isolated callus tissue was extracted using Trizol reagent (catalog 15596018, Invitrogen) following the manufacturer’s instructions and was reverse-transcribed into cDNA with iScript cDNA Synthesis Kit (catalog 170-8890, Bio-Rad). qRT-PCR was performed with TB Green Premix Ex Taq II Kit (catalog RR82WR, TaKaRa) with primers listed in Supplemental Table 1.

For SSC and Treg bulk-RNA-Seq analysis, 1,000 fresh SSCs or Tregs from callus tissue of Treg-depleted mice and control mice 7 days after surgery and from BM tissue at steady state were collected by FACS. Total RNA was then extracted for generating sequencing libraries by using the TruSeq RNA Sample Prep Kit (catalog RS-122-2001, Illumina). The barcoded samples were pooled and then sequenced on an Illumina Hiseq platform, and 125 bp/150 bp paired-end reads were generated. The RNA-Seq library generation and sequencing were performed at Novogene Corporation. A heatmap was used to display relative transcript levels of genes of interest by using normalized fragments per kilobase million (FPKM) values from Cuffnorm. A volcano plot was generated by ggplot2 package in R. The pathway gene sets used in this work were extracted from the online databases Kyoto Encyclopedia of Genes and Genomes (KEGG) (https://www.genome.jp/kegg/), GO (https://geneontology.org/), BioCarta (https://www.hsls.pitt.edu/obrc/index.php?page=URL1151008585), Reactome (https://reactome.org/), and curated Hallmark gene sets (https://docs.gsea-msigdb.org/#MSigDB/Release_Notes/MSigDB_2023.2.Hs/). Function terms or pathways were considered significant if Benjamini–Hochberg adjusted FDR < 0.05.

### Bioinformatics analysis of scRNA-Seq data.

For analysis of scRNA-Seq data from BM of human patients with fracture, fracture nonunion, and from normal BM, raw FASTQ data was derived from PRJNA900553 and then was extracted into a Seurat object. Quality control was processed by filtering out these cells: (a) gene numbers less than 200, unique molecular identifiers (UMI) less than 1,000, and log10GenesPerUMI less than 0.7; (b) more than 10% of the counts belonged to mitochondrial genes and more than 5% of the counts belonged to hemoglobin genes. Quality control revealed no significant batch effect, and similar distributions were observed for the metrics mentioned above across different runs and experiments. Detailed procedures are described in Supplemental Methods.

### ATAC-Seq and analysis.

ATAC-Seq was performed according to the Omni-ATAC protocol (52) without modifications. A preamplification of transposed nuclear fragments was performed using primers with Illumina adaptors. Barcoded sample libraries were pooled for a final concentration of 4 nM. For sequencing data analysis, the adapters were trimmed and aligned to the mm10 reference genome by Bowtie2. Reads mapped to mitochondrial DNA, with duplication or unknown identities were eliminated by Sambamba. The read depth was 80 to 90 million reads for each sample. The total number of mapped reads in each sample was normalized to one million mapped reads. Peak calling was performed using MACS285 with a *q* value cutoff of 0.01. The peaks were assigned to each gene locus, including 20 kb upstream of the transcription start site, gene body, and 5 kb downstream of the transcription termination site. For visualizing the ATAC-Seq data, bigwig files were created from bam files with deeptools, normalized using the counts per million mapped reads (CPM) method (53), and then the peaks were visualized in Integrative Genomics Viewer (IGV, version 2.10.3). SitePro (version 1.0.2) was used to visualize the average signals of ATAC-Seq in the desired genomic regions. Homer findMotifsGenome.pl (version 4.8.3, homer de novo Results) was used to identify transcription factor motifs enriched at peaks.

### CCL1, CCR8 inhibitor, and CCL1 inhibitor treatment.

For CCL1 administration in vivo, recombinant mouse CCL1 protein (50 μg/kg, catalog 845-TC, R&D) in 50 μL of LPS-free PBS was injected intraperitoneally after defect surgery and this was repeated every 3 days until sacrifice. To interrupt the CCL1-CCR8 axis, CCR8 inhibitor (1 mg/kg, catalog ML604086, MedChemExpress) or CCL1 inhibitor (75 μg each, catalog AF845, R&D) was administered intraperitoneally after defect surgery and this was repeated every 3 days (for CCR8 inhibitor) or 7 days (for CCL1 inhibitor) until sacrifice. The mice in the control group were treated with an identical volume of corn oil. For monitoring the role of CCL1 in regulating the activity of BATF and PGRN secretion in Tregs, Tregs were sorted from SP and lymph node and cultured as described above. CCL1 (10 ng/ml, 50 ng/ml) was added to the culture medium after 2 hours of culture. After 24 hours, cells were collected for flow cytometry analysis.

### Treg transfection.

For Treg transfer experiments, Tregs were freshly isolated from SPs of 8- to 10-week-old male WT and *Grn*^–/–^ mice using EasySep Mouse CD4^+^CD25^+^ Regulatory T Cell Isolation Kit II (catalog 18783, STEMCELL Technologies) as above. The isolation was performed in a 2-step procedure with a negative selection on CD4^+^ cells and a positive selection on CD25^+^ cells according to the manufacturer’s instructions. DT treatment was given every 2 days after surgery. Then, 2–3 × 10^6^ Tregs diluted in 50 μL sterile PBS were injected into recipient *Foxp3*-DTR mice 3 days after DT treatment through the lateral tail vein, which was repeated every 7 days. Control mice received an equivalent volume of PBS. For PGRN treatment, recombinant mouse PGRN protein (50 μg/kg, catalog HY-P74617, MedChemExpress) was administered intraperitoneally after defect surgery, which was repeated every 3 days until sacrifice.

### Dual luciferase assay.

Transfection for dual-luciferase reporter plasmids was performed as described above. Luciferase activity was measured using the Dual Luciferase Reporter Assay System (catalog E1910, Promega) according to the manufacturer’s instructions. In brief, HEK293A cells were transfected with the indicated plasmids using Lipofectamine 2000 (catalog 11668027, Thermo Fisher Scientific). At 48 hours after transfection, cells were lysed by passive lysis buffer and lysate was harvested. The firefly and renilla luciferase activity in the lysate were detected in triplicate using a dual-luciferase reporter assay system (Promega) according to the manufacturer’s protocol and quantified using the GloMax 20/20 luminometer (Promega). The luciferase activity of firefly was normalized to renilla luciferase activity to determine the target gene promoter activity. Luciferase activity of promoter was expressed as fold changes of control group.

### In vitro suppression assay.

Teff cells (CD4^+^CD25^−^GFP^–^ cells) were sorted and labeled with CellTrace Violet Cell Proliferation dye (catalog C34557, Thermo Fisher Scientific) according to the manufacturer’s instructions and were used as responder cells. Then labeled Teff cells (1 × 10^5^) were sorted and cocultured with *Grn^–/–^* or WT Tregs in the presence of 1 μg/ml soluble anti-CD3/CD28 in 96-well U-bottom plates. The ratios of Tregs to Teff cells were 0:1, 1:1, 1:2, 1:4, 1:8, or 1:16. After 72-hour incubation, the proliferation of Teff cells was determined by flow cytometry as previously reported (54).

### Statistics.

Statistical analyses were performed using GraphPad Prism, version 6.0, with unpaired, 2-sided Student’s *t* test for 2-group comparisons. One-way ANOVA or 2-way ANOVA with Bonferroni’s multiple-comparisons test was used for multiple groups. All data are shown as mean ± SEM. The numbers of experimental repeats are shown in figure legends. A *P* value of less than 0.05 was considered to be significant.

### Study approval.

All mouse experiments complied with all relevant ethical regulations and were performed according to protocols approved by the Institutional Animal Care and Use Committees at the Shanghai Jiao Tong University and in compliance with the ARRIVE 2.0 guidelines (55).

### Data availability.

RNA-Seq data and ATAC-Seq data have been deposited in the Sequence Read Archive under accession no. PRJNA863720, PRJNA863747. The published human scRNA-Seq dataset used in this study is accessible at the NCBI’s Gene Expression Omnibus (GEO PRJNA900553). The Batf Chip seq data is accessible at GEO (GSE39756). The binding sites of BATF within the *Grn* promoter were predicted using the JASPAR database (https://jaspar.genereg.net/). The paper and the supplementary material present all data needed to evaluate the conclusions. Values for all data points in graphs are reported in the Supporting Data Values file. Other data generated or analyzed during this study are available from the authors upon request.

## Author contributions

JS, HL, and RC conceived the project. RC provided the methodology. RC, XZ, YY, YL, B Liu, SQ, and YG carried out the investigations. HC, B LI, QW, KM, JS, and HL provided the resources. RC, XZ, and HC carried out the formal analysis. XZ and HC curated the data. JS and HL supervised the project. RC and JS wrote the original draft of the paper. MST, JS, and HL edited and revised the paper. RC is listed as the first author in recognition of her significant contribution to the inception of this study.

## Supplementary Material

Supplemental data

Supplemental table 1

Supplemental table 2

Supporting data values

## Version 1 11/07/2024

In-Press Preview

## Version 2 01/16/2025

Electronic publication
