# Supplementary material for: Progranulin-dependent repair function of regulatory T cells drives bone-fracture healing
Source: J Clin Invest. 2024 Nov 7;135(2):e180679. doi: 10.1172/JCI180679 (PMC11735098; doi:10.1172/JCI180679)
Supplement: Supplemental data [file jci-135-180679-s012.pdf]

Supplemental information

Supplemental methods

Bioinformatics analysis of single-cell RNA sequencing data

For analyzing the scRNA-seq data, we used functions from Seurat for downstream analysis. “NormalizeData” followed by the “ScaleData” function was used to normalize and scale the sequencing reads. Top 2000 highly variable genes were identified by “FindVariableGenes” function for PCA analysis. Cells were clustered using the “FindClusters” function and visualized using a 2-dimensional Uniform Manifold Approximation and Projection (UMAP) algorithm with the Run UMAP function. For high-resolution analysis of Treg cell cluster, this subset was extracted, renormalized, and reintegrated as above using 2000 variable features. cells were subclustered in the same workflow. Wilcoxon rank sum test was used to find differentially expressed genes (DEGs) as compared to other clusters. ‘‘FindMarkers’’ function was used to identify DEGs between Treg1 cluster and Treg2 cluster. P value < 0.05 and $|log2foldchange| > 0.58$ was set as the threshold for significantly differential expression. ClusterProfiler R package was used to identify significantly enriched GO terms and KEGG pathways. Violin plots displaying the gene expression were generated by Vlnplot function. We also performed the pseudotime-trajectory analysis for Treg cells subclusters using Monocle3. The trajectory was then projected to the same UMAP embedding generated by Seurat during cell clustering analysis. To identify possible cell–cell communication, CellChat was run on four CTR and six FTD-GRN donors according to the tutorial called ‘Comparison analysis of multiple

datasets' on their GitHub page. The aggregated interaction numbers and weights between different cell types in Treg1 cluster and Treg2 cluster were generated as two circle plots using 'netVisual_circle' function in CellChat, with edge weights scaled to be comparable between Treg1 cluster and Treg2 cluster. The major sources and targets for all cell-cell communications within a cell population were generated as a scatter plot using an adapted version of 'netAnalysis_signalingRole_scatter' function in CellChat. Contribution of each ligand-receptor pair to the overall signaling pathway was visualized by 'netAnalysis_contribution' function in CellChat. The conserved and context-specific signaling pathways between PMF and control were identified and visualized using 'rankNet' method in CellChat.

Supplementary Figures

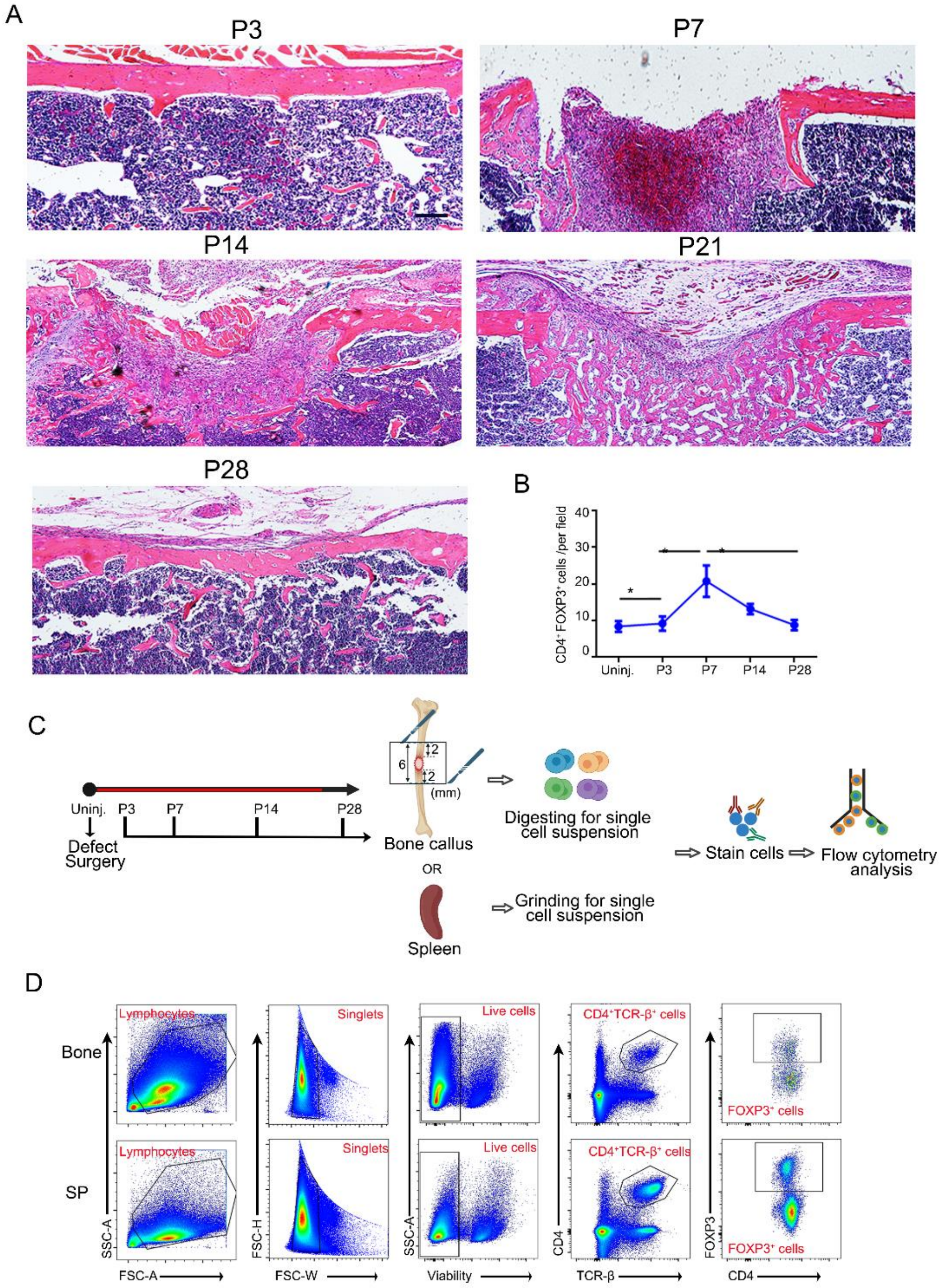

Supplemental Figure 1. Immunofluorescence staining and flow cytometry analysis of Treg cells expansion at the injury site.

(A) Representative HE staining images of uninjured bone tissue (Uninjury) and injured bone tissue on day 3, day 7, day 14, and day 28 post-operation. Scale bars: 200μm.

(B) Quantitative analysis of $CD4^{+}FOXP3^{+}$ cells in each field by using Image J. n= 5 per group. All data are shown as mean ± s.e.m. *P ⩽ 0.05, as determined by one-

way ANOVA with Bonferroni multiple comparisons test.

(C) Schematic strategy to observe the Treg cells proportion and number within the injury site or spleen at indicated time points.

(D) Representative flow cytometry analysis images of BM and SP Treg cells.

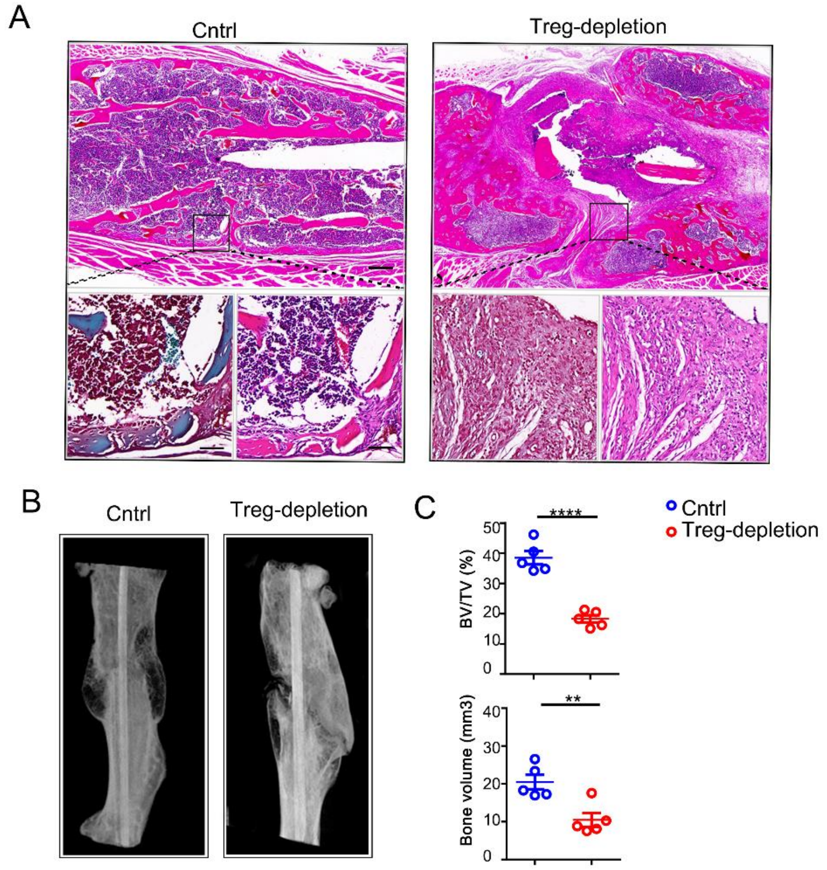

Supplemental Figure 2. Treg-depletion impairs bone fracture healing.

(A) Representative HE staining (Upper and Lower right panel) and Safranin O staining (Lower left panel) of fractured tissue from Control (Cntrl) and Treg-depletion group 28 days after fracture. Scale bars: 500 µm (Upper); 25 µm (Lower).

(B) Representative micro-CT images showing fracture healing.

(C) Quantitative analysis of bone volume fraction (BV/TV) and bone volume of callus tissue. n=5 per group.

All data are shown as mean ± s.e.m. $**P \leq 0.01$, $****P \leq 0.001$, as determined by unpaired two-tailed Student's t-test.

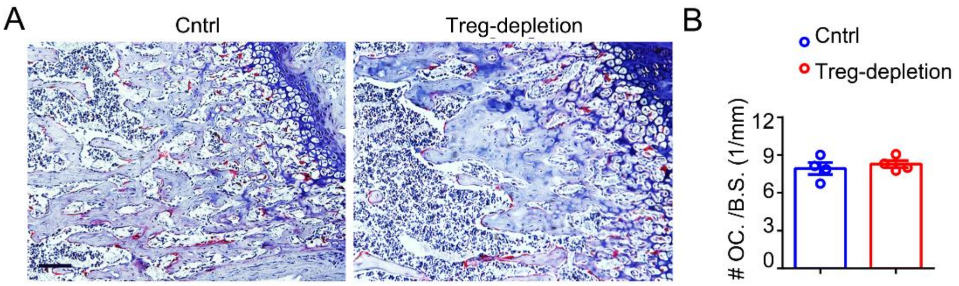

Supplemental Figure 3. TRAP staining analysis of the influence of Treg cells depletion on osteoclasts activity.

(A) Representative histological images of femur bone stained with TRAP in mice from control group and Treg-depletion group. Scale bar = 50 μm.

(B) Statistical analysis of TRAP-positive cells in histological sections (n = 4). Data are shown as mean ± s.e.m, as determined by unpaired two-tailed Student's t-test.

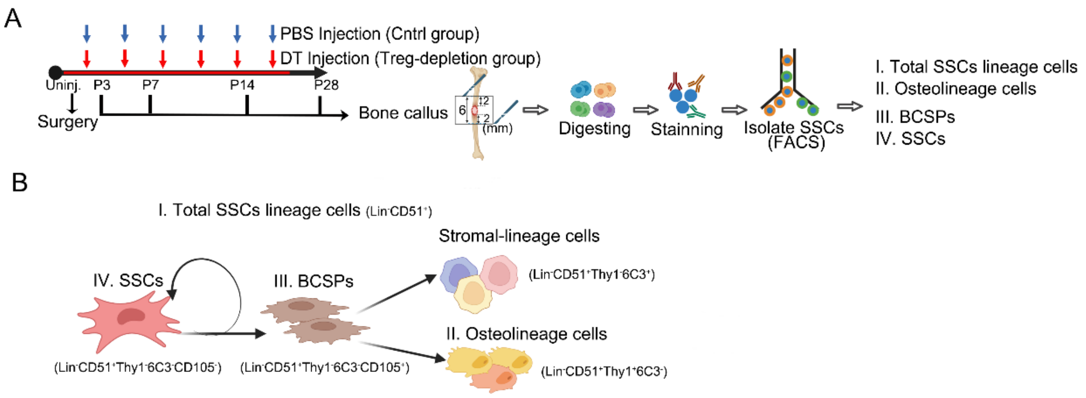

Supplemental Figure 4. Diagrammatic images showing the isolation and differentiation of SSC lineage cells.

(A) Diagrammatic illustration of the creation of Treg cell-depleted mice model with bone injury and the isolation of SSCs from bone callus tissues.

(B) Diagrammatic illustration of the SSCs lineage cells differentiation.

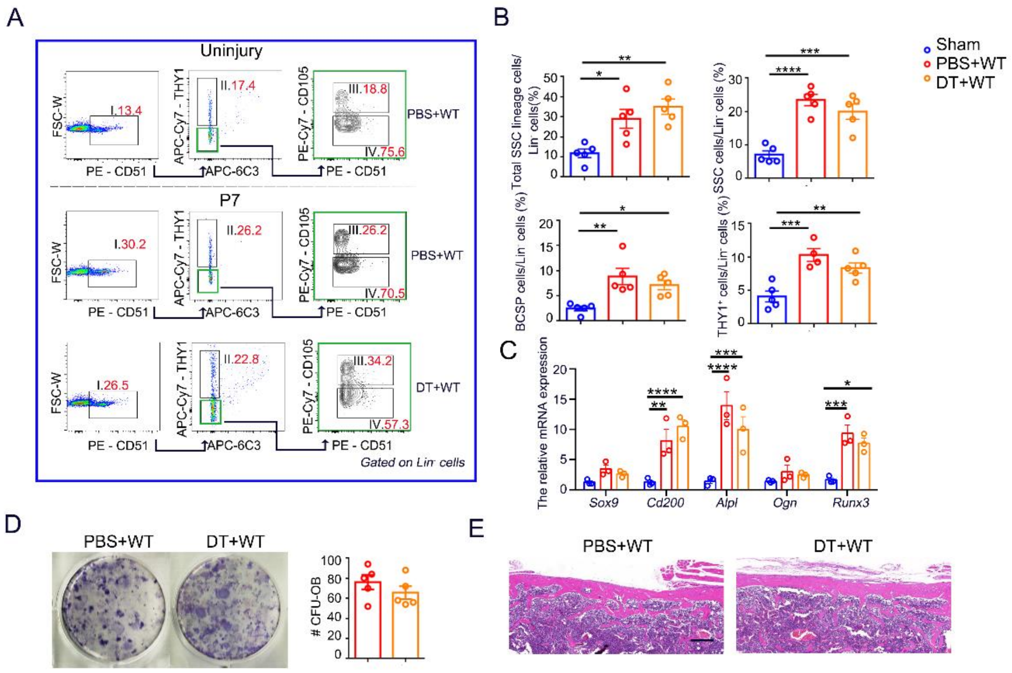

Supplemental Figure 5. DT influences in SSCs and bone healing.

(A) Representative flow cytometry images of SSC lineage cells in the PBS treated and DT treated group.

(B) The percentages of total SSC lineage cells, SSC, BCSP, and Thy1$^+$ cells within total Lin$^-$ cells from PBS/DT treated group on P7 compared with uninjured mice (Sham). n =5 per group.

(C) Transcript levels of osteogenic genes in SSCs isolated from the injury site in PBS and DT treated group on P7 compared with SSCs from the uninjured mice. n =3 per group.

(D) Quantification of colonies of osteogenic (CFU-OB) assays showing the differentiation results of SSCs in PBS and DT treated group. n = 5 per group.

(E) HE staining of injured bone tissues from the PBS and DT treated group on day 28 after surgery. Scale bar: 200 um.

All data are represented as mean ± s.e.m. *P ≤ 0.05, **P ≤ 0.01, ***P ≤ 0.005, ****P ≤ 0.001, as determined by one-way ANOVA with Bonferroni multiple comparisons test (B), two-way ANOVA with Bonferroni multiple comparisons test (C) or unpaired two-tailed Student’s t-test (D).

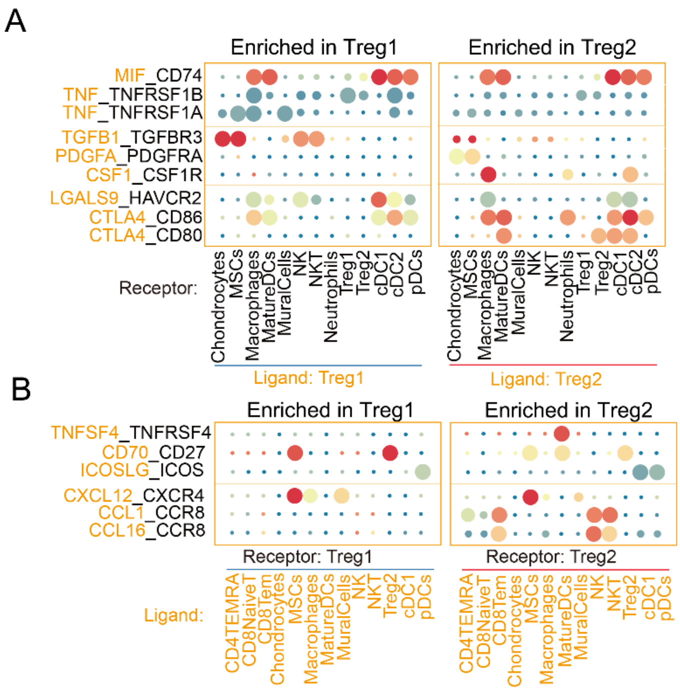

Supplemental Figure 6. Visualization of the selected Treg 1/2- bone marrow cells crosstalk pathways.

(A) Schematic showing Treg 1/2- bone marrow cells crosstalk pathways in which ligands were expressed in Treg1/2 clusters.

(B) Schematic showing Treg 1/2- bone marrow cells crosstalk pathways in which

receptors were expressed in Treg1/2 clusters.

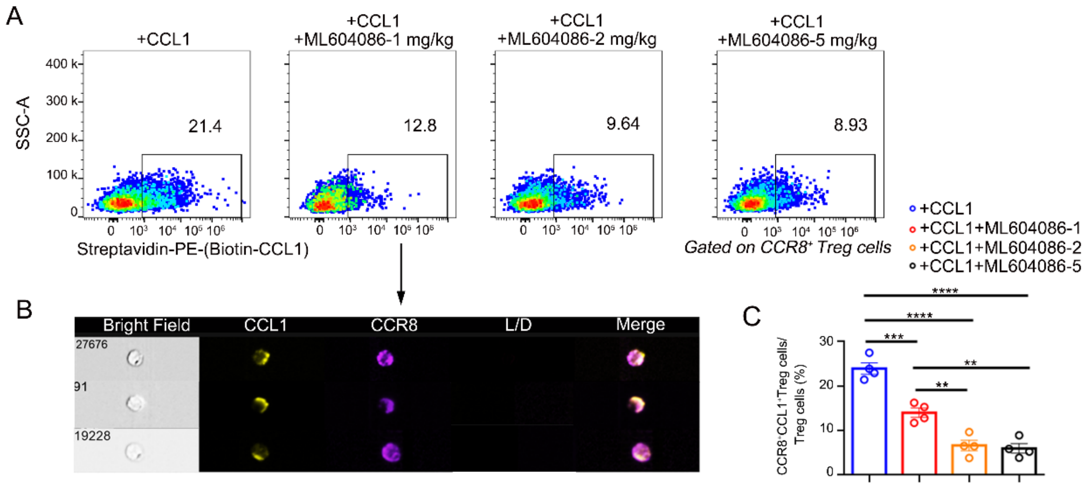

Supplemental Figure 7. ML604086 treatment inhibit CCL1-CCR8 binding.

(A) Representative flow cytometry images of $CCR8^{+}CCL1^{+}$ cells in CCL1 or CCL1+ ML604086 treated groups.

(B) Treg cells after cultivation with CCL1-biotin were analyzed by ImageStream syste. Expression of CCL1-biotin and CCR8 in $CCR8^{+}$ Treg cells was confirmed at the single cell level.

(C) Percentages of $CCR8^{+}CCL1^{+}$ cells within $CCR8^{+}$ cells. n = 4 per group.

All data are represented as mean ± s.e.m. $^{**}P \leq 0.01$, $^{***}P \leq 0.005$, $^{****}P \leq 0.001$, as determined by one-way ANOVA with Bonferroni multiple comparisons test.

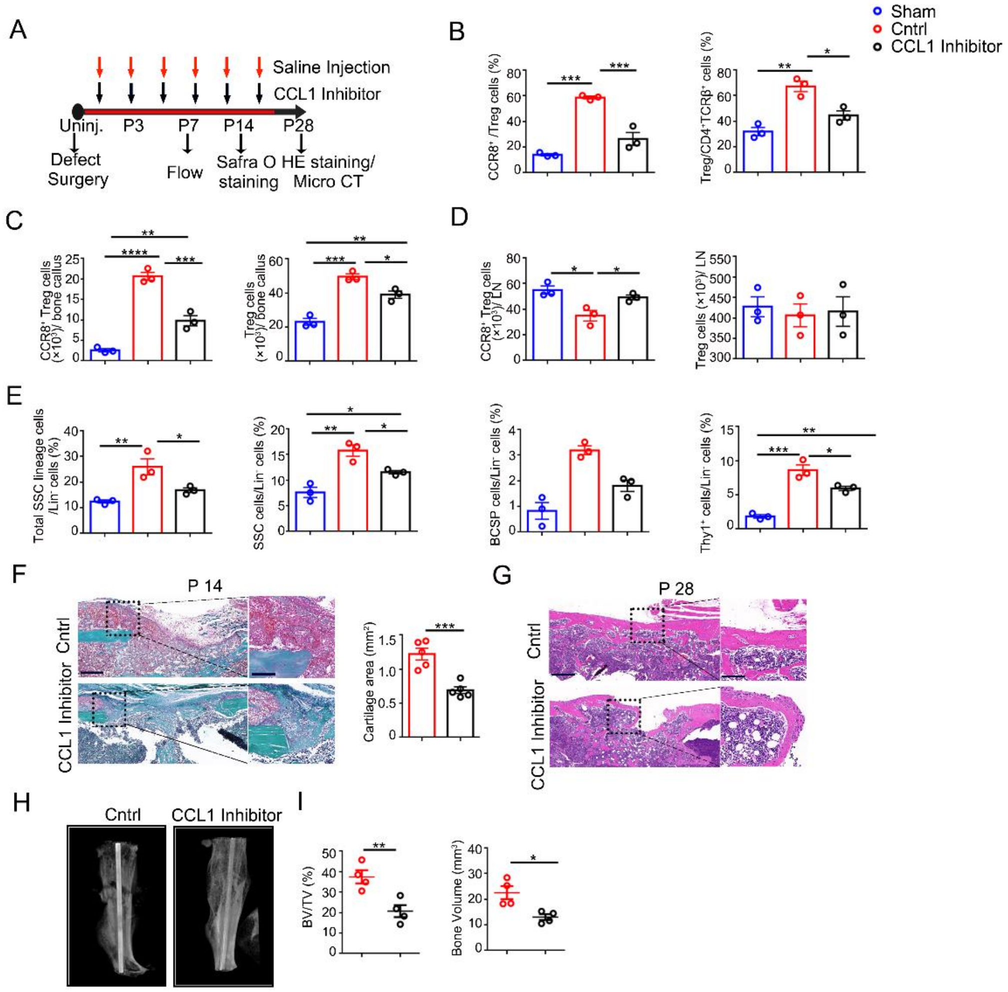

Supplemental Figure 8. CCL1-Ab impairs bone healing through blocking CCL1-CCR8 axis.

(A) Schematic diagram showing the CCL1-Ab treatment protocol.

(B) Proportion of $CCR8^+$ Treg cells among Treg cells and Treg cells among $CD4^+TCR\beta^+$ cells from uninjured group (Sham, blue) and from injury site in control group (Cntrl, red), CCL1-Ab treated group (CCL1-Ab, black) on seven days after surgery. n = 3 per group.

(C) The numbers of $CCR8^+$ Treg cells and total Treg cells in bone callus. n = 3 per group.

(D) The numbers of $CCR8^+$ Treg cells and total Treg cells in adjacent inguinal lymph node (LN). n = 3 per group.

(E) Frequencies of total SSC lineage cells, the SSCs, the BCSP cells, and the $Thy1^+$ cells in Lin^- cells. n = 3 per group.

(F) Safranin O staining images (Left) of bone tissues at injury site on day 28 after injury and quantification analysis of safranin O images (Right). n=5 per group.

(G) HE of bone tissues at injury site on day 28 after injury. Scale bars: 200μm (Left); 50 μm (Right).

(H) Representative micro-CT images demonstrate fracture healing.

(I) Micro-CT quantification of bone volume fraction (BV/TV) and bone volume of callus tissue. n=4 per group.

All data are shown as mean ± s.e.m. *P ≤ 0.05, **P ≤ 0.01, ***P ≤ 0.005, ****P ≤ 0.001, as determined by one-way ANOVA with Bonferroni multiple comparisons test (B-E) or unpaired two-tailed Student's t-test (F, I).

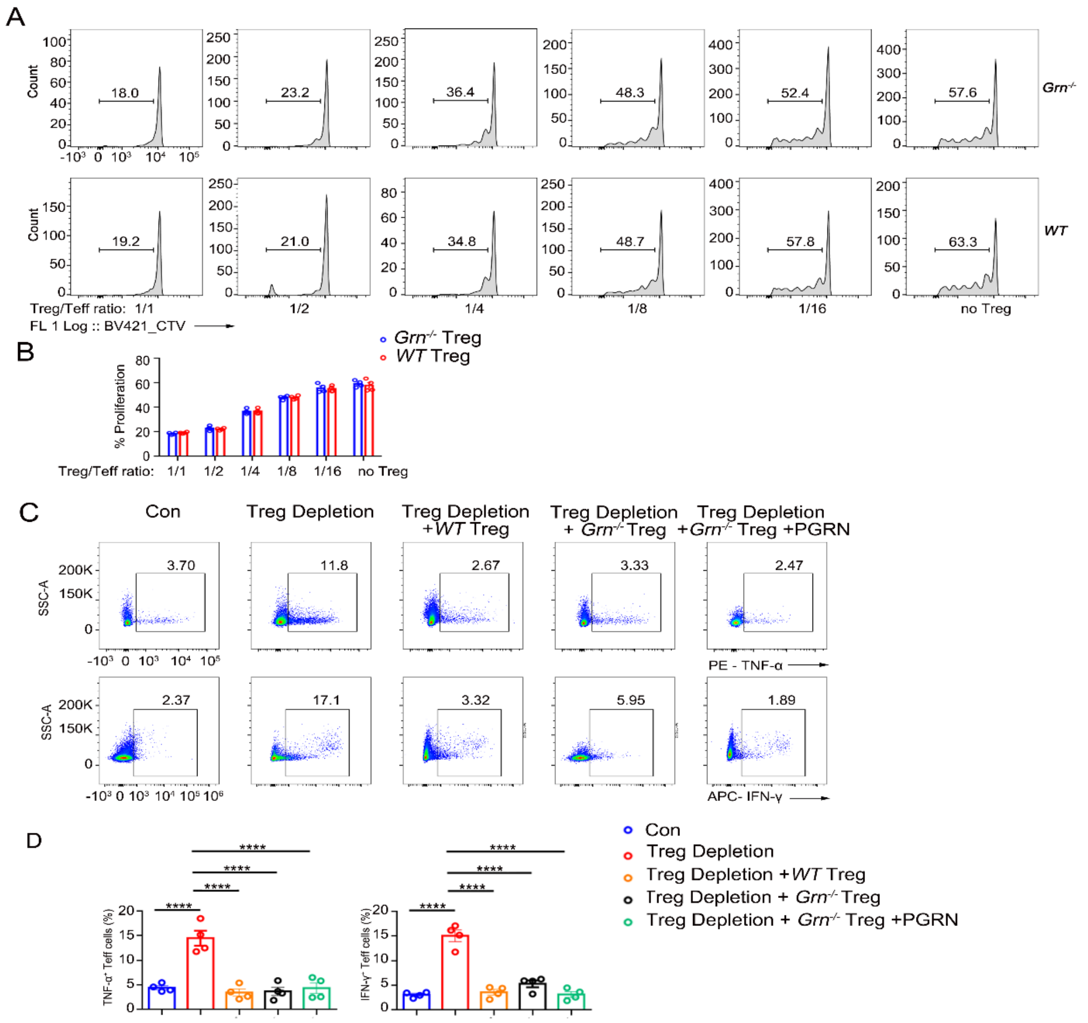

Supplemental Figure 9. *Grn* deficiency does not affect the immunosuppressive function of Treg cells.

(A) Representative flow cytometry analysis images showing the proliferation of Teff cells.

(B) Quantification of the CFSE positive Teff cells. n = 4 per group.

(C) Representative flow cytometry analysis of IFN-γ^+ and TNF-α^+ Teff cells from callus tissue on day 7 post-operation.

(D) The proportions of IFN-γ^+ and TNF-α^+ Teff cells among Teff cells. n = 4 per group. All data are represented as mean $\pm$ s.e.m. ****P $\leqslant$ 0.001, as determined by

two-way ANOVA with Bonferroni multiple comparisons test (B) or one-way ANOVA with Bonferroni multiple comparisons test (D).

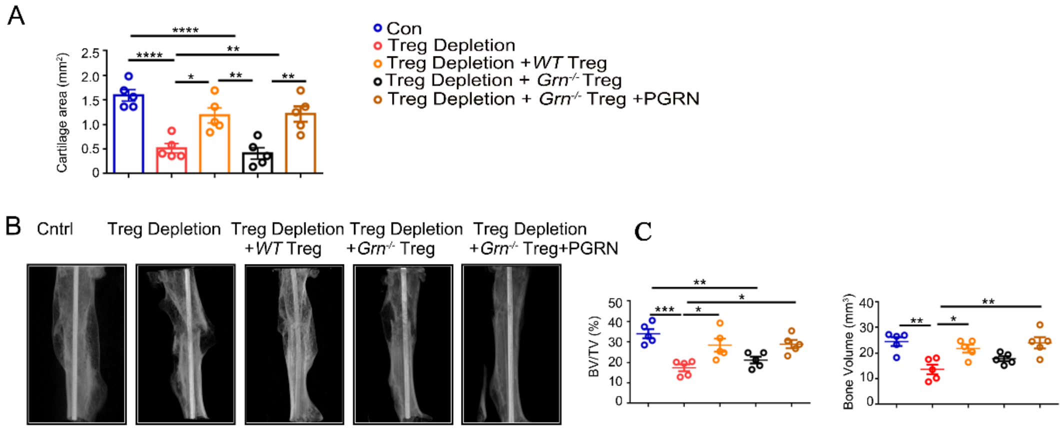

Supplemental Figure 10. PGRN deficiency impairs the bone repair role of Treg cells.

(A) The Safranin O images were analyzed using Image J Ver.1.48. n = 5 per group.

(B) Representative micro-CT images showing fracture healing.

(C) Quantitative analysis of bone volume fraction (BV/TV) and bone volume of callus tissue. n = 5 per group.

All data are represented as mean ± s.e.m. *P ≤ 0.05, **P ≤ 0.01, ***P ≤ 0.005, ****P ≤ 0.001, as determined by one-way ANOVA with Bonferroni multiple comparisons test.

Supplemental Table 1. The antibody information.

Name	**Antibody (Clone number)**	**Company**	**Catalog number**	**Dilutions**
CD4	Anti-CD4-APC (clone GK1.5)	Biolegend	Cat# 100412	1:200;
Fixable Viability Dye	Fixable Viability Dye eFluor™ 780	eBioscience	Cat# 65-0865-14	1:1000;
TCRβ	Anti-TCRβ-PE-Cy7 (clone H57-597)	Biolegend	Cat# 109221	1:200;
FOXP3	Anti-Foxp3-FITC (clone FJK-16s)	eBioscience	Cat# 11-5773-82	1:200;
CD200	Anti-CD200-BV650 (clone OX-90)	BD biosciences	Cat# 745402	1:100;
CD105	anti-CD105-PE-CY7 (clone MJ7/18)	Biolegend	Cat# 120410	1:200;
6C3	anti-CD6c3-APC (clone 6C3)	Biolegend	Cat# 108312	1:200;
THY1.1	anti-THY1.1-APC-CY7 (clone HIS51)	ebioscience	Cat# 47-0900-82	1:200;
THY1.2	anti-THY1.2-APC-CY7 (clone 53.21)	ebioscience	Cat# 47-0902-82	1:200;
CD45	anti-CD45-Percp-CY5.5 (clone 30-F11)	Biolegend	Cat# 103132	1:200;
CD31	anti-CD31-Percp-CY5.5 (clone 390)	Biolegend	Cat# 102420	1:200;
TER119	anti-TER119-Percp-CY5.5 (clone TER-119)	Biolegend	Cat# 116228	1:200;
CD51	anti-CD51-PE (clone RMV-7)	BD biosciences	Cat# 551187	1:50;
CD44	anti-CD44-PE (clone REA664)	Miltenyi Biotec	Cat# 130-118-566	1:200;
CD62	anti-CD62L-APC (clone MEL14)	Biolegend	Cat# 104412	1:200;
CTLA	anti-CTLA-APC (clone UC10-4B9)	eBioscience	Cat# 17-1522-822	1:200;
CCR8	anti-CCR8-BV421 (clone SA214G2)	Biolegend	Cat# 150305	1:100;
KLRG1	anti-KLRG1-PE-CY7 (clone 2F1)	eBioscience	Cat# 25-5893-82	1:200;
PGRN	anti-PGRN	Abcam	Cat# ab187070	1:100
BATF	anti-BATF-PE	CST	Cat# 27120	1:200;

CD25	anti-CD25-PE (clone 3C7)	BD biosciences	Cat# 553075	1:200;
GFP	anti-GFP	ABclonal	Cat# AE102	1:500
CD16/CD32	BD Pharmingen™ purified rat anti-mouse CD16/CD32 antibody	BD Pharmingen	Cat# 553141	1:50;
CCL1	Anti-CCL1 Polyclonal Antibody	Bioss	Cat# Bs-10710R	1:100
CCL1	CCL1 Neutralizing Antibody	R&D	Cat# AF845	
F4/80	F4/80 Monoclonal Antibody (BM8)	Invitrogen	Cat# 14-4801-82	1:200
IFN-γ	APC anti-mouse IFN-γ Antibody (XMG1.2)	Biolegend	Cat# 505809	1:200
TNF-α	PE anti-mouse TNF-α Antibody (MP6-XT22)	Biolegend	Cat# 506305	1:200
	rabbit anti-mouse IgG Isotype control	Abcam	Cat# ab172730	1:100
	Alexa Fluor 488-conjugated goat anti-mouse IgG	Invitrogen	Cat# A32723	1:500;
	Alexa Fluor 647-conjugated goat anti-rabbit IgG	Invitrogen	Cat# A32733	1:500;
	Streptavidin-PE	Invitrogen	Cat# EPX-SAPE-000	1:1000

Supplemental Table 2. The Sequences of RT–qPCR primers.

Primer Name	Sequence
CCL1-F	5'-GCTTACGGTCTCCAATAGCTGC-3'
CCL1-R	5'-GCTTTCTCTACCTTTGTTCAGCC-3'
CCL2-F	5'-GCTACAAGAGGATCACCAGCAG-3'
CCL2-R	GTCTGGACCCATTCCTTCTTGG-3'
CCL17-F	5'-CGAGAGTGCTGCCTGGATTACT-3'
CCL17-R	5'-GGTCTGCACAGATGAGCTTGCC-3'
CCL8-F	5'-GGGTGCTGAAAAGCTACGAGAG-3'
CCL8-R	5'-GGATCTCCATGTACTCACTGACC-3'
CCL22-F	5'-GTGGAAGACAGTATCTGCTGCC-3'
CCL22-R	5'-AGGCTTGCGGCAGGATTTTGAG-3'
CCL4-F	5'-5'-ACCCTCCCACTTCCTGCTGTTT-3'
CCL4-R	5'-CTGTCTGCCTCTTTTGGTCAGG-3'
GAPDH-F	5'-TGTGTCCGTCGTGGATCTGA-3'
GAPDH-R	5'-TTGCTGTTGAAGTCGCAGGAG-3'
GRN-Mut-F	5'-CAACTCAGAACTTGATCCCTGCC-3'
GRN-Mut-R	5'-TTTCCCTAAGTCCCGTTCAATCC-3'
GRN-WT-F	5'-ATGTACAGCACCATCTATGAGCTA-3'
GRN-WT-R	5'-TTTCCCTAAGTCCCGTTCAATCC-3'
